# Simultaneous defeat of MCF7 and MDA-MB-231 resistances by a hypericin PDT–tamoxifen hybrid therapy

**DOI:** 10.1038/s41523-019-0108-8

**Published:** 2019-04-10

**Authors:** Theodossis A. Theodossiou, Muhammad Ali, Mantas Grigalavicius, Beata Grallert, Pierre Dillard, Kay Oliver Schink, Cathrine E. Olsen, Sébastien Wälchli, Else Marit Inderberg, Andreas Kubin, Qian Peng, Kristian Berg

**Affiliations:** 1 Department of Radiation Biology, Institute for Cancer Research, Radium Hospital, Oslo University Hospital, Montebello, 0379 Oslo, Norway; 2 Department of Immunology, Institute for Cancer Research, Radium Hospital, Oslo University Hospital, Montebello, 0379 Oslo, Norway; 3 Department of Cellular Therapy, Department of Oncology, Radium Hospital, Oslo University Hospital, Oslo, Norway; 4 Department of Molecular Cell Biology, Institute for Cancer Research, Radium Hospital, Oslo University Hospital, Montebello, 0379 Oslo, Norway; 5 PLANTA Naturstoffe Vertriebs GmbH, A-1120 Wien, Austria; 6 Department of Pathology, Radium Hospital, Oslo University Hospital, Montebello, 0379 Oslo, Norway

## Abstract

Currently the greatest challenge in oncology is the lack of homogeneity of the lesions where different cell components respond differently to treatment. There is growing consensus that monotherapies are insufficient to eradicate the disease and there is an unmet need for more potent combinatorial treatments. We have previously shown that hypericin photodynamic therapy (HYP-PDT) triggers electron transport chain (ETC) inhibition in cell mitochondria. We have also shown that tamoxifen (TAM) enhances cytotoxicity in cells with high respiration, when combined with ETC inhibitors. Herein we introduce a synergistic treatment based on TAM chemotherapy and HYP-PDT. We tested this novel combinatorial treatment (HYPERTAM) in two metabolically different breast cancer cell lines, the triple-negative MDA-MB-231 and the estrogen-receptor-positive MCF7, the former being quite sensitive to HYP-PDT while the latter very responsive to TAM treatment. In addition, we investigated the mode of death, effect of lipid peroxidation, and the effect on cell metabolism. The results were quite astounding. HYPERTAM exhibited over 90% cytotoxicity in both cell lines. This cytotoxicity was in the form of both necrosis and autophagy, while high levels of lipid peroxidation were observed in both cell lines. We, consequently, translated our research to an in vivo pilot study encompassing the MDA-MB-231 and MCF7 tumor models in NOD SCID-γ immunocompromised mice. Both treatment cohorts responded very positively to HYPERTRAM, which significantly prolonged mice survival. HYPERTAM is a potent, synergistic modality, which may lay the foundations for a novel, composite anticancer treatment, effective in diverse tumor types.

## Introduction

All scientific efforts to find a cure for cancer stumble across one obstacle, simple yet difficult to circumvent: cancerous cells come from random mutations of normal cells, in an effort to escape the tight controls imposed on them. These include their metabolism, the way they feed, the rate at which they proliferate and their defenses against controlled death or the immune system professional killers, among other homeostatic parameters.^1,2^ This leads to the formation of cancers which are unique and also quite heterogeneous, since they are derived from many generations of cells. This heterogeneity is the main reason why monotherapies are likely to fail as universal cancer treatment, since one part of the tumor could strongly respond to this treatment while other parts could exhibit a certain degree of tolerance to the monotherapy. In contrast, combinatory treatments can simultaneously target many of the differential weaknesses, across a panel of cancer cell lines, so that the combo-treatment can then be applied as universally as possible, without the need of prescreening for efficacy.

MCF7 and MDA-MB-231 cells represent a striking example in that they are both invasive ductal/breast carcinoma cells, yet they have many phenotypic/genotypic differences: MCF7 are hormone dependent (both estrogen and progesterone receptor positive—ER and PR), while MDA-MB-231 are triple negative. The lack of ER has rendered MDA-MB-231 insensitive to treatments with antiestrogens, such as the selective estrogen receptor modulator tamoxifen,^3^ which is widely used in breast cancer chemoprevention,^4–6^ but also as an adjuvant to primary disease.^7,8^ Metabolically, MCF7 cells are more Pasteur type relying on ATP production from oxidative phosphorylation at normoxic conditions but increase their glycolytic activity under hypoxia, while MDA-MB-231 cells are more Warburg type, mainly relying on glycolysis for ATP production under both normoxic and hypoxic circumstances.^9,10^ Finally MCF7 cells express the epithelial phenotype in contrast to MDA-MB-231 that are more mesenchymal^11^ and have also been documented for their multidrug resistance.^12^

Photodynamic therapy of cancer, PDT,^13,14^ provides the most selective cancer treatment through the synergy of three essential, yet individually non-chemotoxic components: (i) the photosensitizer (PS), i.e. a light activated drug; (ii) light of the appropriate wavelength to excite the PS, and (iii) oxygen being the terminal generator of toxic species upon interaction with the excited PS.^15,16^ Consequently, the photodynamic action is effected through the generation of reactive oxygen species (ROS) either by (i) charge transfer which could involve oxygen superoxide anion and hydrogen peroxide ultimately leading to the formation of hydroxyl radicals^17^ (type I mechanism) or (ii) energy transfer, leading to the production of deleterious singlet oxygen [O_2_ (¹Δ_g_) or ^1^O_2_] (type II mechanism).

The main limitation of PDT is the penetration depth of light, which in tissue can, in the best-case scenario, reach a few millimeters. Nevertheless, in clinical PDT, apart from superficial application of light for cutaneous lesions, there is also the possibility to administer light to lesions in hollow organs (e.g. the esophagus) endoscopically, using side illuminating fiber optics or interstitially, for inner solid organs, with the use of spinal needles through which the front illuminating fiber optics are fed to reach the lesion. In this later case, several treatment stations can be achieved to cover bigger lesions, by pulling back the spinal needle under radiological guidance (CT, MRI, or ultrasound).

In our previous work^18^ we established mechanistically why the two adenocarcinoma cell lines MDA-MB-231 and MCF7 have differential responses to hypericin photodynamic therapy (HYP-PDT). MDA-MB-231 cells exhibit vulnerability to HYP-PDT and concomitant membrane lipid peroxidation, due to their lack of the membranic glutathione peroxidase (GPX4),^18^ while on the other hand MCF7 lack the xenobiotic detoxification enzyme Glutathione *S* transferase (GSTP1)^18^ which facilitates the expulsion of toxic xenobiotics from the cell through the GS-X pump.^19,20^ In this context, possibly also due to their lack of GSTP1, the estrogen-receptor-positive MCF7 cells exhibit increased vulnerability to the specific estrogen receptor modulator tamoxifen (TAM) due to its genomic effect.^3,21,22^ Elsewhere, we additionally demonstrated that MCF7 cells were also vulnerable to TAM non-genomically due to their enhanced respiratory metabolism.^23^ This non-genomic TAM-induced MCF7 cytotoxicity was shown to be exacerbated by inhibition of the quinoloxidizing center of mitochondrial respiratory chain complex III.^23,24^ Combining this with our earlier finding that HYP-PDT causes an irreversible inhibition of cytochrome *c* reduction at the quinoloxidizing center of complex III,^25^ we hypothesized that a combinatorial treatment of HYP-PDT and an acute TAM administration would simultaneously target the MDA-MB-231 PDT vulnerability and the MCF7 “Achilles’s heel”, i.e. TAM, complementing the HYP-PDT effects.

In the present project, we evaluated the combination of HYP-PDT and TAM administration (HYPERTAM) to compare how the combinatorial treatment affected the two cell lines in vitro but also in vivo in animal MCF7 and MDA-MB-231 tumor models and whether there were any synergistic effects in either of the two cell lines. Such a treatment combination could be used for breast cancer irrespective of their hormone dependence.

## Results

### Subcellular localization of TAM and HYP

Initially we looked at the co-localization of TAM with HYP in the two chosen cell lines by employing the FITC-labeled NDMTAM-FITC. Representative micrographs are shown in Fig. S1. The micrographs reveal a close localization of HYP and NDMTAM-FITC in MDA-MB-231 cells, with NDMTAM colocalizing very well with Mitotracker® Deep Red both in MCF7 and MDA-MB-231 cell lines (Fig. S2). The colocalisation of HYP with NDMTAM-FITC is less apparent, especially in MCF7 cells, as seen in Fig. S1. However, MCF7 cells exhibit much higher respiratory activity^23^ and could possibly be significantly turning HYP into its non-fluorescent hydroquinone,^26^ especially since we have shown that HYP can act as a substrate at complex III of the ETC.^25^ Due to close proximity of HYP to cell mitochondria as well as previous repeated documentations of mitochondrial action of HYP-PDT,^25,27–29^ indirectly indicating close proximity, we decided to apply the HYP-PDT and 4-OHT combinatorial treatment which we call “HYPERTAM strategy”.

### Synergistic effects of HYPERTAM on MCF7 and MDA-MB-231 cells using MTT

**Fig. 1:**
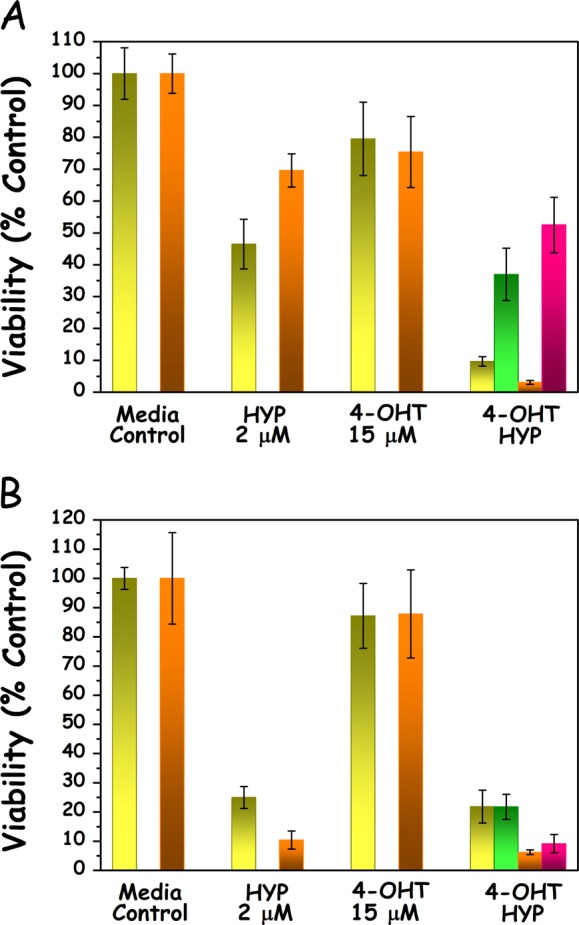
The effects of HYP-PDT and 4-OHT combinatorial treatment (HYPERTAM). **a** MCF7 and **b** MDA-MB-231 cells. The MTT-derived viabilities are shown in yellow for 24 h post-irradiation and orange for 48 h post-irradiation. The calculated HYPERTAM 24 h viability values by extrapolation from the HYP-PDT and 4-OHT mono-treatments appear in green. The corresponding 48 h calculated values appear in pink. A HYPERTAM synergistic effect is only evident in MCF7 cells at these timeframes. The data shown are representative from one of at least three replicate experiments. The error bars represent STD values on eight parallels

The results of the HYPERTAM strategy are shown in (Fig. 1). In both cell lines the MCF7 HYP-PDT LD_50_ light dose was applied and in both cell lines the HYPERTAM strategy caused profound photo-chemotoxicity as reflected in the 24 and 48 h MTT assays. In the case of MCF7 (Fig. 1a) HYP-PDT induced a 50% reduction in viability at 24h after treatment, which was reduced to ~30% at 48 h probably due to cell re-proliferation. 4-OHT (15 μM) monotherapy, on the other hand, caused a moderate reduction in viability of ~20–25% both at 24 and 48 h following administration. The combinatory treatment (HYPERTAM) however instigated a staggering ~90% reduction in viability at 24 h which became ~97% at 48h following treatment. These enhanced photochemotoxicities of HYPERTAM did not correspond to the additive effects of HYP-PDT and 4-OHT individual toxicities which were calculated according to the formula:

1 $${\mathrm {HYPERTAM}\,{\mathrm {viability}}}\,\left( \% \right) = \frac{{{\mathrm {HYP}}\mbox{-} {\mathrm {PDT}}\,{\mathrm {viability}}\,\left( \% \right) \times 4 \mbox{-} {\mathrm {OHT}}\,{\mathrm {viability}}\,(\% )}}{{100\,(\% )}}.$$

These calculated additive viabilities for the HYPERTAM strategy are 37% and 52% for MCF7 cells for 24 and 48 h post-treatment, respectively; however, the corresponding experimental values were merely 9.5% and 3% (*p* < 0.00001). In the case of MDA-MB-231 (Fig. 1b) the experimental and calculated values were in very good agreement, indicating additive effects. HYP-PDT affected quite a profound phototoxicity, 75% at 24h and ~90% at 48 h post-irradiation. 4-OHT administration had only a mild toxicity effect on the cells, i.e. ~15% both at 24 and 48 h post-administration. The HYPERTAM toxicities, 78% at 24h and 94% at 48 h, are in line with the additive toxicities of HYP-PDT and 4-OHT monotherapies as calculated from Eq. (1).

The ratio of the experimental over the calculated HYPERTAM survival values was determined for MCF7 and MDA-MB-231 cells using at least four independent experiments. These ratios were found to be

$$\left( {\frac{{{\mathrm {HYPERTAM}}_{\mathrm{exp}}}}{{{\mathrm{HYPERTAM}}_{{\mathrm {calc}}}}}} \right)_{{\mathrm {MDA} {\mbox{-} {\mathrm {MB}} \mbox{-} 231}}} = 1.06 \pm 0.16\,{\mathrm {and}}\,\left( {\frac{{{\mathrm{HYPERTAM}}_{{\mathrm {exp}}}}}{{{\mathrm {HYPERTAM}}_{{\mathrm {calc}}}}}} \right)_{{\mathrm {MCF7}}} = 0.20 \pm 0.17\,(p = 0.00012)$$

These ratios indicate that in the case of MDA-MB-231 cells the experimental survival values in average agree with the calculated values, suggesting a purely additive effect, while for MCF7 cells the experimental survival values are in average 20% the calculated ones suggesting a strong synergistic effect. This is in line with our hypothesis that HYP-PDT irreversibly inhibits mitochondrial complex III,^25^ thus profoundly exacerbating the cytotoxic action of tamoxifen, similarly to the effects of tamoxifen combination with the complex III inhibitor, myxothiazol.^23,24^ Even though in MDA-MB-231 cells there was not a synergistic effect like the one in MCF7 cells, the viability at 48h (6%) greatly decreased in comparison to that at 24h (~22%) showing no signs of recovery for the cells after the combinatory assault. Similarly, the viability of MCF7 cells was reduced from ~9.5% at 24h to ~3% at 48 h post-treatment. These results indicate that the HYPERTAM strategy was successful in defeating the mutually exclusive resistances (to tamoxifen and PDT, respectively) of the two cell lines and conferring acute cytotoxicity.

### Assessment of HYPERTAM efficacy on MCF7 and MDA-MB-231 cells using the clonogenic assay

**Fig. 2:**
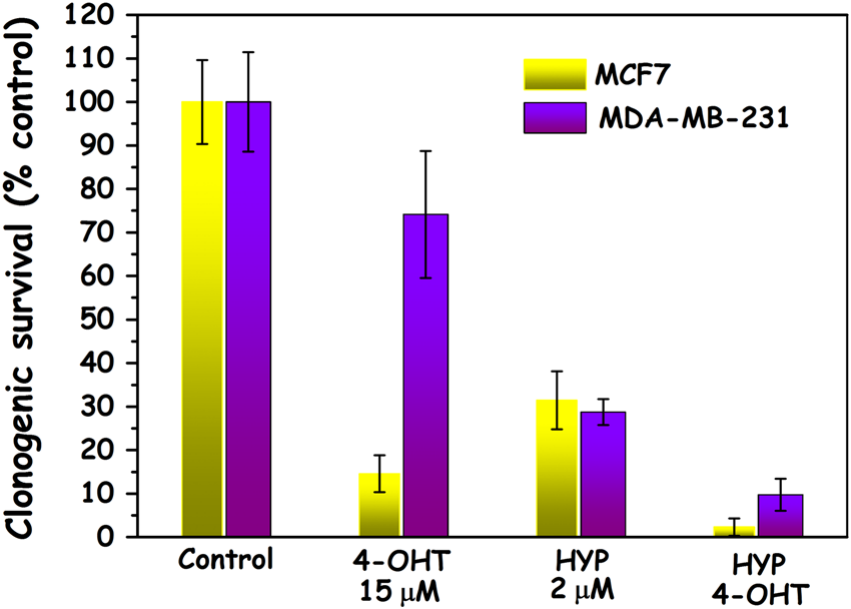
Clonogenic survival following HYPERTAM. Results of clonogenic assays in MCF7 (yellow) and MDA-MB-231 cells (blue-purple). The data shown are representative from two replicate experiments. The error bars represent STD values on 2–3 parallels

Next, we investigated the clonogenicity of the various treatment groups. The results are shown in Fig. 2. As expected, the MCF7 cells were found to be considerably more vulnerable to 4-OHT treatment (~15% survival) than MDA-MB-231 cells (~75% survival). The slightly reduced survival of the MDA-MB-231 cells is most likely due to the well-documented non-genomic toxicity of tamoxifen^21,23,30–32^ Even though MDA-MB-231 are more responsive to HYP-PDT, in Fig. 2 both cell lines responded similarly to PDT, because the light doses were adjusted to their respective HYP-PDT susceptibilities.^18^ Nevertheless, both cell lines exhibited quite profound responses to the combined HYP-PDT and 4-OHT i.e. HYPERTAM treatments (MCF7 ~3% survival, MDA-MB-231 ~9% survival). We repeated the HYPERTAM clonogenicity experiments in MCF7 cells that had their estrogen receptors (both ESR1 and ESR2) knocked down (by 60% and 40%, respectively) and the results are shown in Fig. S3. From these data it is evident that knocking down the ESR in MCF7 cells profoundly abrogated both the 4-OHT and HYPERTAM long-term toxicities. These two respective MCF7 survivals in the case of ESR1,2 knock down (Fig. S3) resemble more the clonogenicities of the corresponding MDA-MB-231 groups (Fig. 2). In parallel experiments using MTT, however, this abrogation was hardly noticeable at 24 h (data not shown).

### Mode of death in MCF7 and MDA-MB-231 cells following HYPERTAM

In order to elucidate the prevalent death mechanisms in the treatment groups (4-OHT, HYP-PDT, and 4-OHT+HYP-PDT), we assessed apoptosis by two different flow cytometry-based assays (Annexin V and TUNEL assay), necrosis by measuring intracellular lactate dehydrogenase (LDH) leakage and autophagy by immunoblotting for LC3-B.

From the flow cytometry plots in Fig. S4 depicting Annexin V vs. LIVE/DEAD IR, we can see that in neither MCF7 cells nor MDA-MB-231 cells we can distinguish a high level of early apoptosis in any of the time points studied (≤10%) except in MDA-MB-231 HYP-PDT group 24 h post-irradiation where Annexin V-positive and LIVE/DEAD IR-negative population reached 16%. It should be noted that in the groups where 4-OHT was used the live populations were shifted to more Annexin V-positive values. This can be attributed to an effect of 4-OHT on cell membranes which has been documented before,^33,34^ probably resulting in a low-level exposure of phosphatidylserine to Annexin V, not necessarily linked to apoptosis. In these cases, it can be seen that although shifted, there is only one intact viable cell population without any distinguishable apoptotic offshoots. Also, necrosis for the HYPERTAM group in both the cases of MCF7 and MDA-MB-231 cells is in accordance with the LDH assay data (vide infra), i.e. reaching 85–90% at 24 h.

Since we could not detect late apoptosis with Annexin V we also performed the TUNEL assay at 8 and 24 h. The resulting data are shown in Fig S5. From these plots we can see that no noticeable apoptotic fractions were detected 8 h post-treatment for any of the treatment groups [CTRL, 4-OHT, HYP-PDT (HYP) or HYPERTAM (HT), less than 3% in apoptotic activity]. In the 24 h groups we saw low levels of late apoptosis in the HYPERTAM groups of both cell lines (~17% in MCF7 and ~7% in MDA-MB-231 cells). In all other groups the apoptotic (TUNEL-stained) fraction remained below 3.5%.

In order to assess necrosis we employed the LDH leakage assay at two time points, namely at 6 and 24 h following the HYP-PDT treatment. The trends at the two time points were very similar, and the results obtained at 24 h are shown in Fig. S6. HYP-PDT instigated negligible necrosis in both cell lines (6% MCF7 and 3% MDA-MB-231), while 4-OHT was found to cause significant necrosis (~40% MCF and ~55% MDA-MB-231). Our initial concern was whether HYP-PDT inactivated LDH during the irradiation process; however, the HYP-PDT+4-OHT (HYPERTAM) curve shows substantial activity of LDH released in the supernatant, corresponding to ~55% and ~75% necrosis for the MCF and MDA-MB-231 cells, respectively. This means that, even though the HYP-PDT regimen did not cause necrosis to the cells, the primary mode of death for 4-OHT and hence also for HYPERTAM was necrosis.

Subsequently we measured the level of autophagy in the various treatment groups employing immunoblotting for the light chain three (LC3) protein cleavage. A representative set of blots for all treatment groups is shown in Fig. S7A. The blots in Fig. S7 reveal low-level basal autophagy in both cell line media controls, which is however profoundly increased both in the 4-OHT and the HYP-PDT treatment groups. Moreover, in the combined 4-OHT+HYP-PDT treatment the LC3-B levels raise at least to the additive level of the two cases. It should be noted that pretreatment with bafilomycin, a potent inhibitor of vacuolar-type H^+^-ATPase and hence positive LC3B control, showed that the LC3B levels in each of the HYP, 4OHT, and HYPERTAM groups had already reached almost maximal levels of autophagy 6 h after treatment.

Collectively, in the case of HYP-PDT autophagy is the most prominent mechanism of cell death, while in the case of 4-OHT and HYPERTAM treatment necrosis and autophagy are the main mechanisms.

### Lipid peroxidation measurements following HYPERTAM

**Fig. 3:**
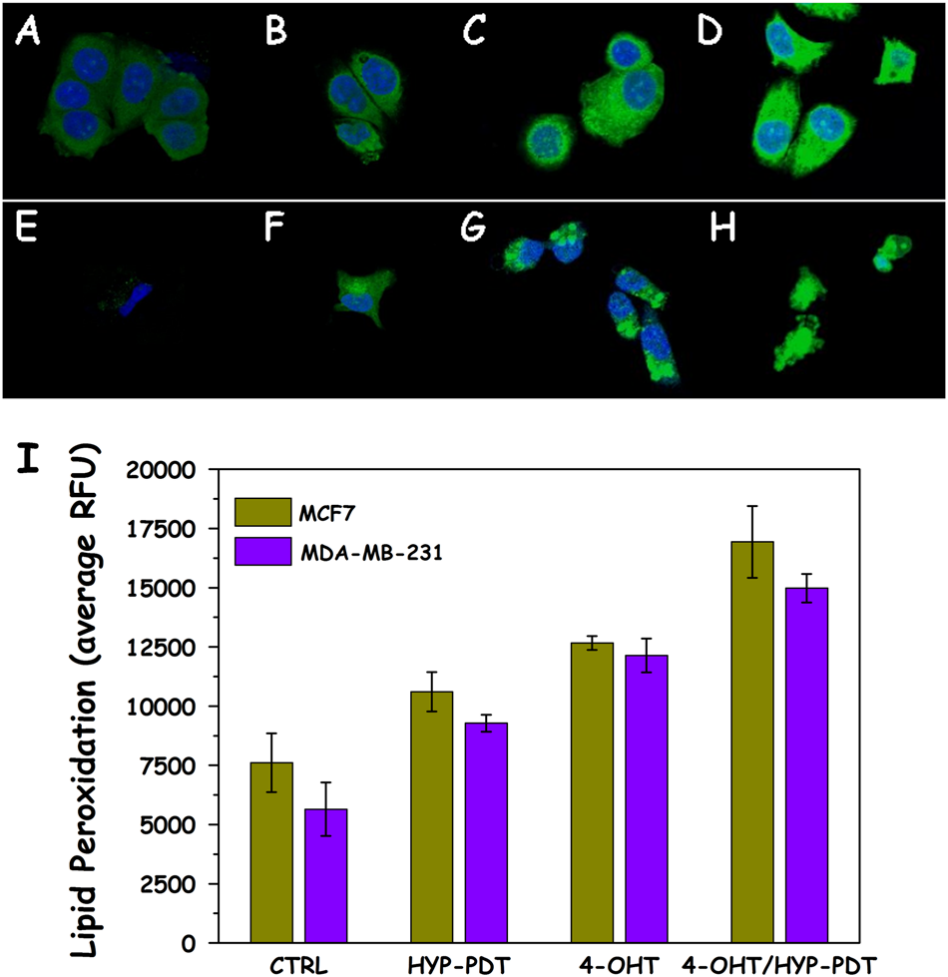
Lipid peroxidation studies following HYPERTAM. Representative confocal microscopy images of cells treated with the lipid peroxidation assay and imaged 6 h post-irradiation. **a**–**d** MCF7 cells and **e**–**h** MDA-MB-231 cells. **a**, **e** Media controls, **b**, **f** HYP (2 μM)-PDT-treated cells, **c**, **g** 4-OHT (15 μM)-treated cells, and **d**, **h** HYPERTAM (HYP-PDT and 4-OHT)-treated cells. Cell nuclei are stained blue with DAPI while lipid peroxidation increases with increasing green fluorescence intensity. **i** Quantitative analysis of the confocal microscopy images. At least eight regions of interest (ROIs) were used in each case, the average of which are presented with their standard deviations as errors. All treatment groups are significantly different from the controls (*p* < 0.00001, by Students two-tailed *t*-test)

We next evaluated the extent of lipid peroxidation in the various treatment groups in the two cell lines. Representative images are presented in Fig. 3. From these it can be seen that there is increased lipid peroxidation in both cell lines (green fluorescence intensity and extent) upon application of HYP-PDT, TAM, and TAM+HYP-PDT, and secondly that the intensity and range of lipid peroxidation in MCF7 control cells (Fig. 3a) is much higher than in MDA-MB-231 control cells (Fig. 3e). This can be attributed to the higher respiratory activity of MCF7, which triggers the generation of ROS even in the absence of an oxidative assault. The differences in lipid peroxidation between the controls and treatment groups are much higher in the case of MDA-MB-231. This can be accounted for by the lack of GPX4 in MDA-MB-231 cells and thus their inability to detoxify lipid peroxides in contrast to the “Pasteur” MCF7 cells.^18^ The results of the analysis of the confocal imaging are averaged and summarized in Fig. 3i, where it is shown that in both cases there is a progressive increase of lipid peroxidation in the order HYP-PDT<4-OHT<HYP-PDT+4-OHT. The values in all treatment groups are significantly different from the controls with 10^−10^ ≤ *p* ≤ 10^−5^, when using a Student’s two-tailed *t*-test, between samples with unequal variance. Moreover, when subtracting the controls’ lipid peroxidation mean value from these of the treatment groups, it is evident that in the case of MCF7 cells the lipid peroxidation of HYPERTAM (~9300 ± 800 RFU) is slightly higher that the additive values of HYP (~3000 ± 250 RFU) and 4-OHT (~5100 ± 120 RFU), whereas in the case of MDA-MB-231 cells the effect seems to be additive (9400 ± 380 vs. 3600 ± 140 and 6500 ± 380 RFU, respectively).

### Metabolic effects of HYPERTAM

**Fig. 4:**
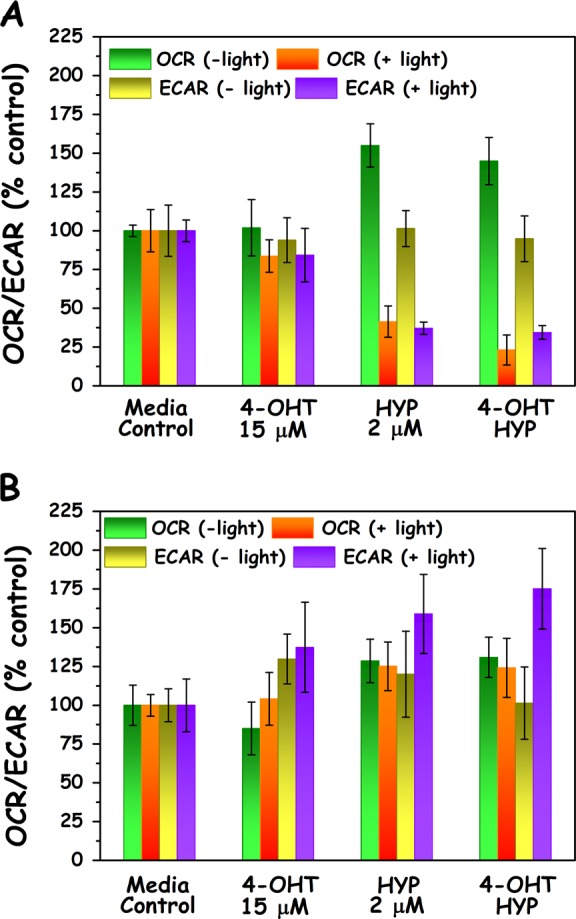
Metabolic analyses following HYPERTAM. Seahorse analyzer-mediated metabolic HYPERTAM studies in **a** MCF7 cells and **b** MDA-MB-231 cells. Oxygen consumption rate (OCR) reflects the respiratory activity of the cells, while extracellular (media) acidification rate reflects the cell glycolytic activity. All values were normalized to DNA content by post-assay use of PicoGreen ® DNA stain

Next, we looked at the metabolism of the treatment groups before and after the application of light for the two cell lines. The results are summarized in Fig. 4. For MCF7 cell the oxygen consumption rates and media acidification rates for media controls and 4-OHT-treated cells were comparable within experimental error and showed no statistically significant deviations. Upon addition of HYP (or HYP + 4-OHT) however and without light irradiation the oxygen consumption rates significantly increase approximately by a factor of 1.5 with respect to the corresponding media control values (CTRL vs. HYP *p* = 0.0003, CTRL vs. HYP-4OHT *p* = 0,0007). This effect was only found in MCF7 cells and not in MDA-MB-231 cells (CTRL vs. HYP *p* = 0.45, CTRL vs. HYP-4OHT *p* = 0.2). The media acidification rates however, which reflect the glycolytic activity, remained at the media control levels prior to irradiation. These results are very significant as they show that HYP caused a considerable increase of respiratory activity in MCF7 cells. This is consistent with our previous results,^25^ where we showed that HYP is a natural substrate for the mitochondrial electron transport chain complex III, at half the *V*_max_ of its natural substrate Q_10_. Addition of HYP most probably assisted the efficient transport of electrons (at least in complex III) enhancing and supplementing the function of Q_10_ and thus increasing oxygen consumption. Half an hour following irradiation however, both the oxygen consumption and media acidification rates of the HYP and HYP+4-OHT treatment groups profoundly decreased to less than 50% these of media control. This signifies the early onset of an HYP-PDT-induced impairment of the cell metabolism, both respiratory and glycolytic, resulting in a bioenergetic collapse.

Conversely, in MDA-MB-231 cells the introduction of HYP (or HYP+4-OHT) to the cells seems to have a slight, marginally significant increase in the oxygen consumption (*p* ~ 0.04), which however does not seem to be affected by the application of light. This is in line with the much lower oxygen consumption rates of the MDA-MB-231 cells in comparison to the MCF7 cells (~1:4), evident of the Warburg phenotype of the former. The media acidification rates however seem to increase considerably upon irradiation for the HYP and HYP + 4-OHT treatment groups, suggesting a distressed increase of the glycolytic rates. In MDA-MB-231 cells there seems to be a slight increase of the acidification rate for the 4-OHT treatment group (±light).

### HYPERTAM studies in animal MCF7 and MDA-MB-231 xenografts

Having established the proof of principle of our hypothesis in cell cultures, we then endeavored to prove our hypothesis also in an in vivo pilot study using immunocompromised NOD SCID-γ (NSG) mice bearing MCF7 and MDA-MB-231 xenografted tumors.

**Fig. 5:**
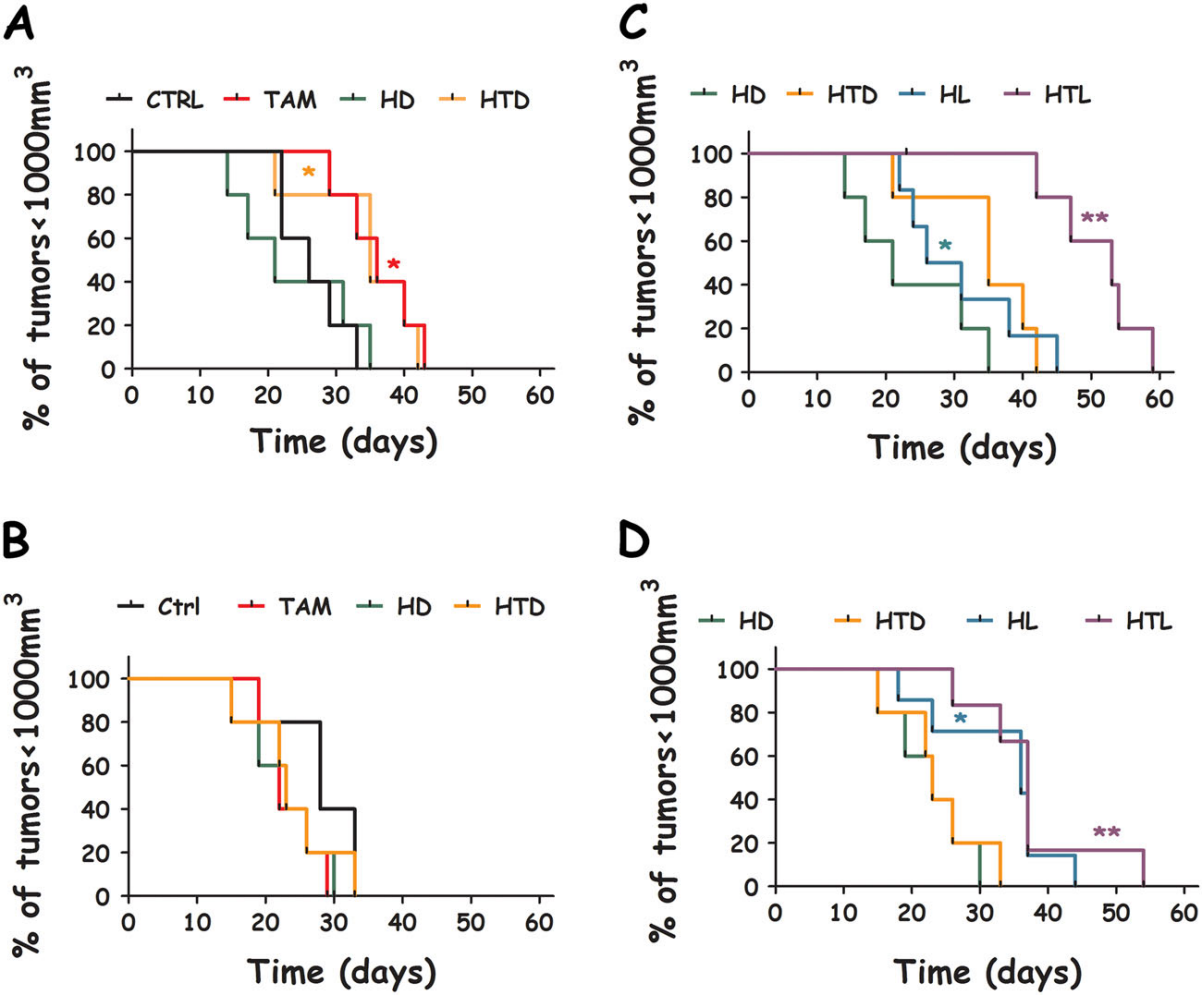
Survival of xenograft-bearing mice following HYPERTAM. NSG mice endpoints (tumor volume ≥ 1000 mm^3^) shown as Kaplan–Meier plots for the different treatment groups of the MCF7 (**a**, **c**) and MDA-MB-231 (**b**, **d**) tumor models. CTRL (untreated), HD (HYP dark), TAM (tamoxifen only), HTD (HYP+TAM dark), HL (HYP-PDT), HTL (HYPERTAM). The error bars represent STD from the mean of seven parallels. The statistical significance was calculated using the log-rank (Mantel–Cox) test; ns *p* ≥ 0.05, *p < 0.05 and **p < 0.005

The treatment-to-endpoint curves (time for the animals to reach the humane endpoint of tumor volume ≤ 1000 mm^3^) are shown in Fig. 5 as survival and the median post-treatment life-span for each group and tumor model are shown in Table S1. The tumor growth curves are shown as normalized percentages of the initial tumor volumes, which is set to 100% (Fig S8). From these data several interesting observations can be made:

1. In the case of the MCF7 tumor model one single bolus of tamoxifen (200 mg/kg) rendered the time-to-endpoint of the TAM and HTD groups significantly longer than those of the CTRL and HD groups (*p* ~ 0.02, Fig. 5a). This was not the case for the MDA-MB-231 tumor model, where CTRL, HD, HTD, and TAM survival curves are not statistically different from one another (*p* ~ 0.1, Fig. 5b).
2. In MCF7 tumors the treatment-to-endpoint curves of the irradiated group HL was not statistically significant from the dark control (HD, *p* = 0.2, Fig. 5c) while the survival curve of the HYPERTAM group (HTL) was significantly different from its corresponding dark controls (HTD, *p* = 0.004, Fig. 5c). In the MDA-MB-231 tumor models, the irradiated group HL was statistically significant from the dark control (HD, *p* = 0.025, Fig. 5d) and even more so was the treatment-to-endpoint curve of the HYPERTAM group (HTL) vs. its corresponding dark control (HTD, *p* = 0.008, Fig. 5d).
3. From the median survival values shown in Table S1 we calculated whether the HYPERTAM effect is an additive effect (HL + TAM groups) or synergistic. To facilitate that we employed the equation *I*_S_= (HTL-CTRL)−[(HL-CTRL)+(TAM-CTRL)].^35^ In the equation above, *I*_S_ stands for synergy index while each group acronym designates the corresponding median survival for each group (i.e. the day at which 50% of the group population had to be sacrificed because of exceeding the humane endpoints set). In our case *I*_S_ = 14.5, which is a positive number denoting the synergistic advantage of HYPERTAM in days in our experiment, and also suggest a synergistic effect beyond the additive outcomes of HYP-PDT and tamoxifen monotherapies.

These observations support the in vitro results of this study. Indeed, the tumor growth curves (Fig S8) clearly show that MDA-MB-231 tumors are more vulnerable to HYP-PDT than MCF7 ones, given also the fact that MDA-MB-231 tumors grow in general considerably faster (~1.5×) than the MCF7 ones.

The metadata on the impact of a hypericin–PDT–tamoxifen hybrid therapy on MCF7 and MDA-MB-231 resistances are available.^36^

## Discussion

In the present manuscript we evaluate the in vitro and in vivo efficacy of a hybrid therapy combining HYP-PDT and TAM chemotherapy. The hypothesis for the present study is based on two main observations: (i) our previous work^25^ showing that HYP-PDT inhibits the quinoloxidizing center of mitochondrial complex III and (ii) our other work showing^23,24^ that the use of mitochondrial electron transport chain complex III quinoloxidizing center inhibitor myxothiazol greatly exacerbates TAM cytotoxicity. The above observations suggested that HYP-PDT in conjunction with administration of tamoxifen (HYPERTAM) could increase the cytotoxicity in MCF7 cells as well as increase the survival in MCF7-tumor-bearing animals. At the same time HYPERTAM could also target TAM-insensitive tumors through HYP-PDT. To assist us in exploring our hypothesis, we additionally employed the triple-negative MDA-MB-231 cells, which are in general drug resistant,^12^ but were found to be more susceptible to HYP-PDT than MCF7 cells.^18^ The two cell lines were selected to represent invasive breast carcinoma cells displaying many phenotypic/genotypic differences and to highlight the principle that the combination treatment would be beneficial in case of very different cancer types.

One of the main problems in cancer chemotherapy is the development of drug-resistant tumor cells, a common phenomenon in patients with advanced tumors.^37,38^ This resistance collectively known as multidrug resistance (MDR) is the main cause of chemotherapy failure in cancer treatment. The best understood form of MDR in human cells is attributed to P-glycoprotein (Pgp)^39^ and/or the MDR-associated protein (MRP);^40–43^ both these proteins are resident on the plasma membrane and can expel a broad range of internalized xenobiotics against a concentration gradient.^39,44,45^

A third form of drug resistance associated with several drug types is effected by increased levels of reduced glutathione (GSH) and/or glutathione *S* transferase (GST)^46–50^ via an export carrier, the GS-X pump,^19^ aka “multispecific organic anion transporter” (MOAT).^20^ We have previously shown that indeed this is the type of drug resistance that could be prominent in MDA-MB-231 cells due to their high levels of GST, especially in comparison with MCF7 cells which do not express GST.^18^ Conversely, MCF7 have enhanced expression of the membrane-bound glutathione peroxidase (GPX4), which is absent in MDA-MB-231.^18^ This different use of GSH in the two cell lines matches their diverse metabolic phenotypes: MCF7 cells perform respiratory ATP production at normoxic conditions and also switch to glycolysis under hypoxia, while MDA-MB-231 rely on glycolysis for ATP production in both normoxic and hypoxic circumstances^9,10^ More respiratory Pasteur type MCF7 cells require stronger antioxidant protection from electron transport chain leakages and the inevitable creation of reactive oxygen species (ROS) causing lipid peroxidation.^23^ Warburg-type MDA-MB-231 cells, on the other hand, do not require that high level of antioxidant protection since their respiratory activity is profoundly subdued, and their main use for GSH is to expel harmful xenobiotics from the cell interior through the GS-X pump.

As it follows, PDT defeated the weak antioxidant defenses of MDA-MB-231 and induced a pronounced lipid peroxidation (Fig. 3) even though their respiratory activity is much lower than that of MCF7 (~1:4). This is probably due to the fact that MDA-MB-231 cells’ lack of the antioxidant enzyme GPX4 which can make MDA-MB-231 cells vulnerable to the non-genomic effects of TAM.^21,23,30–32^ 4-Hydroxytamoxifen (4-OHT) on the other hand induced extensive cytotoxicity to estrogen receptor-positive MCF7 cells both genomically (long term) as well as non-genomically, especially enhanced by HYP-PDT -mediated complex III quinoloxidizing center shutdown. In addition to hydroxyl-radical-mediated lipid peroxidation in Pasteur MCF7 cells, redox interactions of tamoxifen quinoid metabolites with complex III result also in toxic tamoxifen semiquinone species formation.^23^

The primary mode of death in both cell lines in response to HYP-PDT did not seem to be either necrosis or apoptosis. Especially at the LD_50_ doses used in the present study there was no necrosis observed through LDH leakage in either of the cell lines for HYP-PDT. However, autophagy was highly exacerbated both in HYP-PDT- and 4-OHT-treated groups. We had previously observed tamoxifen associated autophagy;^23^ however, it was not found to be associated with cytotoxicity. Autophagy is not a bona fide cell death initiator but rather a pro-survival mechanism. In some cases, however, its aberrant stimulation can promote cell death.^51^ Indeed, several studies show that HYP-PDT may induce cell death associated with the aberrant induction of macroautophagy,^52,53^ which in this study could be true for both the MCF7 and MDA-MB-231 cells.

The metabolic studies show the different response of the two cell lines in the HYP-PDT and/or 4-OHT assault. Initially the MCF7 cells increase their respiration rate significantly when incubated with HYP in the dark (Fig. 4a). This however cannot be the reason for the apparent faster tumor growth at early days in the xenograft models (Fig S8), as it can only be observed in the case of the less respiratory MDA-MB-231 cells and not in the case of MCF7 cell. The apparent, higher tumor growth in the case of MDA-MB-231 cells could give reason for concern when considering occult metastases, as the primary lesions would be treated with light. On the other hand, it should be pointed out that it does not significantly affect the survival outcome in the xenograft models (Fig. 5, HD and CTRL). In the future we plan to thoroughly investigate the effects of HYP on a panel of cancer cells, for the translation of our technology. The increased respiration rate of MCF-7 cells can be attributed to the fact that HYP is a reductive substrate of complex III (at the quinoloxidizing center) at ½ *V*_max_ of the complex’s natural substrate coenzyme Q_10_.^25^ The addition of HYP boosts the transport of electrons and hence also enhances the respiratory needs for oxygen. Photoactivation of HYP inactivates complex III,^25^ decreasing the respiratory rate dramatically (Fig. 4b). Apart from the respiratory rate drop, the glycolytic rate also collapses in MCF7 causing an overall metabolic breakdown in these cells. The HYP-PDT-related drop in glycolytic activity in MCF7 is mechanistically unclear, though it could be related to earlier findings that HYP-PDT inhibits the binding of hexokinase (HK) to mitochondria.^29^ HK is bound to mitochondrial porins located in the outer mitochondrial membrane^54^ and regulates the glucose-driven energy supply, as it catalyzes the phosphorylation of glucose for its entry into glycolysis. According to Miccoli et al.,^29^ HYP-PDT induced a local pH drop, causing HK detachment from the outer mitochondrial membrane. In MDA-MB-231 on the other hand, HYP activation does not seem to affect the metabolic profile apart from an increase in the acidification (glycolytic) rate. The differential effects in MDA-MB-231 cells, where glycolytic activity was increased under the HYP-PDT assault, could be attributed to mechanisms protective of their glycolytic function given their altered mitochondrial physiology and/or functions which can only support low ATP turnover from oxidative phosphorylation. This could explain the increase in glycolysis to compensate for the HYP-PDT-induced respiratory stress from the shutdown of electron transport as for example seen after treatment with myxothiazol in both cell lines cells treated with myxothiazol.^23^

The main results of the present in vitro work to be highlighted are those of the clonogenic studies of Fig. 2 showing that the combination of HYP-PDT and tamoxifen (4-OHT) chemotherapy, i.e. HYPERTAM, can simultaneously overcome tamoxifen resistance (through PDT) and PDT resistance through tamoxifen. It is clear from the clonogenicity experiments (after ESR depletion) that the main reason behind the high, long-term toxicity of 4OHT on MCF7 cells (which cannot be seen in MDA-MB-231 cells) is the genomic effect of tamoxifen. The abrogation of this long-term toxicity by ESR depletion is however not so evident in MTT assays at the 24 h timepoint in MCF7 cells (data not shown). We believe that the extent of the restitution of the clonogenic capacity of MCF7 cells depleted for ESR is limited by (i) the transient nature of the knockdown in a comparatively long-term process such as the clonogenic assay and (ii) the low knockdown efficiency (60% and 40% for ESR1 and 2, respectively).

In addition, in MCF7 cells, the HYPERTAM combination resulted in synergistic cytotoxicity within 24 h from light application as revealed by MTT (Fig. 1), presumably due to the inhibition of the quinoloxidizing center of complex III by HYP,^25^ in a similar fashion to application of the selective inhibitor myxothiazol in our previous studies.^23,24^

The difference between the results of the MTT and clonogenic assays can be mainly attributed to the time-lapse between treatment and assay; throughout all our studies with tamoxifen^23,24^ including the present work, we have found that tamoxifen is not very reactive within 24 h of incubation even in MCF7 cells (70–90% survival), when assayed with respect to cell redox functionality (MTT assay). This is very convenient when the aim is to investigate tamoxifen as an adjuvant, synergistic treatment. In the clonogenic assay (Fig. 2) however, where the assay time is significantly longer (~2 weeks) despite the fact that the 4-OHT was removed after 24 h, the ability of cells to make clones is highly reduced in MCF7 cells most probably due to the genomic effect of tamoxifen.^3,55,56^ In the clonogenic assay there is also a toxic effect of 4OHT on MDA-MB-231 cells (less than on MCF7), which we have also encountered in our previous work^23^ and which is probably due to the well-documented non-genomic toxicity of tamoxifen^21,30–32^

Most notably, a single TAM high dose bolus in MCF7 tumors conferred a significant survival benefit vs. untreated controls, as mentioned above. This difference was increased by two and four high boluses of TAM twice weekly increasing survival way beyond the control or that of a single bolus (Fig. S9). In this sense we expect the present study to affect the current philosophy in TAM-based treatments. Instead of small daily doses (20 mg/kg) of tamoxifen, intended to work only on the genomic level, repeated high boluses of 200 mg/kg weekly or twice weekly could trigger both non-genomic cytotoxic effects^23^ and longer term genomic responses. The same stands also for HYPERTAM in MCF7 tumors: most probably repeated high boluses of TAM would also largely benefit HYPERTAM and make it even more efficacious than shown in the present work.

Our study suggests that there is a considerable advantage of HYPERTAM in the MCF7 cells both in vitro and in vivo, as predicted by our hypothesis. A notable advantage in the case of the MDA-MB-231 is probably due to the additive effects of TAM (non-genomic effects) and HYP-PDT. The fact that we had no permanent cures in our HYPERTAM (or any other) animal groups can be attributed to two main factors: (i) the mice that were used (NSG) were completely immunocompromised and thus HYPERTAM did not benefit from an adjuvant immunological effect as it would have in an organism with a fully functional immune system and (ii) due to the low wavelength of excitation of HYP, which does not allow for deep penetration into the tumor tissue. We believe that the proof of principle we have provided in this work could become a viable clinical treatment modality with the choice of another PS, analogous to HYP when considering localization and function, but with a much longer wavelength of activation.

## Materials and methods

### Chemicals and reagents

RPMI 1640 without phenol red, l-Glutamine, penicillin/streptomycin, trypsin, dimethylsulfoxide (DMSO), *N*-desmethyltamoxifen (NDMTAM), 4-hydroxytamoxifen (4-OHT), tamoxifen HCl (TAM), rotenone (ROT), antimycin-A (ANTI-A), oligomycin (OLIGO), carbonyl cyanide 4-(trifluoromethoxy)phenylhydrazone (FCCP), polyethylene glycol sorbitan monolaurate (TWEEN 20), bovine serum albumin (BSA), Triton X-100, thiazolyl blue tetrazolium bromide (MTT), 123 bp DNA Ladder, DNA loading buffer, crystal violet, bafilomycin A1, terminal transferase (TdT kit), biotin-16-dUTP, anti-γ-tubulin and sodium pyruvate were purchased from Sigma-Aldrich Norway AS (Oslo, Norway). ProLong® Gold Antifade Reagent with/without DAPI, Image-iT® FX Signal Enhancer, BlockAid™ blocking solution, Quant-iT™ PicoGreen® dsDNA Reagent, LIVE/DEAD™, dithiothreitol, TopVision agarose, 50× TAE electrophoresis buffer, Streptavidin-Alexa Fluor™ 488 conjugate, Hoechst 33258 pentahydrate, MitoTracker® Deep Red FM, and Click-iT® lipid peroxidation imaging kit-Alexa fluor® 488 were purchased from Thermofisher Scientific (Waltham, MA, USA). GelRed™ was obtained by Biotium Inc. (Hayward, CA, USA). All consumables related to a Seahorse XF^e^96 analyzer were purchased from Seahorse Biosciences Europe (Copenhagen, Denmark). Hypericin (HYP, 99.3%) was obtained from Planta Natural Products GmbH (Vienna, Austria). 17β-Estradiol (E2) was purchased from Cayman Chemical Company (Ann Arbor, MI, USA), Cremophor® EL was obtained from BASF SE (Limburgerhof, Germany), Annexin V-FITC-conjugated was purchased from ImmunoTools GmbH (Friesoythe, Germany), and *N-*desmethyltamoxifen FITC (NDMTAM-FITC) prepared as described elsewhere^24,57^ was kindly donated by Dr. K. Yannakopoulou, INN, NCSR “Demokritos”.

### Cell culture

For the purposes of this study we chose MCF7 and MDA-MB-231 (triple negative) human breast adenocarcinoma cell lines. All cells were originally obtained from ATCC and grown in RPMI 1640 without phenol red, supplemented with 10% fetal bovine serum (FBS), penicillin/streptomycin at 37 °C in a 5% CO_2_ and 95% humidified atmosphere. Cells were inoculated into 96-well plates (2 × 10^4^ cells/100 μL media/well), six-well plates (1 × 10^6^ cells/2 mL media/well), glass bottom 35 mm Petri dishes (Mattek Corp., 1 × 10^5^ cells/2 mL media/dish) 24 h prior to treatment or confocal microscopy imaging.

### Live cell deconvolution imaging

MCF7 and MDA-MB-231 cells were seeded on glass bottom 35 mm dishes (Mattek Corp., 1 × 10^5^ cells per dish) 24 h prior to the experiments. The cells were subsequently treated with (i) 10 μM NDMTAM-FITC 4 h and (ii) 2 μM HYP 4 h. The specific subcellular organelle fluorescent probe for mitochondria (Mitotracker®-Deep Red), was always added to the cells 20 min prior to imaging at a concentration of 150 nM. Cells were subsequently washed with phosphate-buffered saline (PBS) and imaged in fresh 10% fetal calf serum containing RPMI 1640.

Imaging was performed on an OMX V4 instrument equipped with sCMOS cameras and a solid-state light source (GE Healthcare). The system was operated in widefield mode to minimize light dose. Z-stacks covering the whole cell (Z-spacing 125 nm) were acquired. Images were deconvolved and aligned using Softworx software (GE Healthcare).

### Confocal microscopy

Cells were seeded on glass bottom 35 mm Petri dishes (1 × 10^5^ cells per dish) 24 h prior to the experiments. After treatment, fixation, permeabilization and labeling as per the Click-iT® lipid peroxidation imaging kit protocol, the cells were treated with DAPI-containing Prolong® Gold antifade reagent (Life Technologies Inc.). The 4-OHT treatment was in this case performed overnight instead of after irradiation, as the cells were fixed 1 h following irradiation and hence 4 h incubation with 4-OHT post-irradiation was not possible.

The cells were examined with a Zeiss LSM 710 confocal microscope (Carl Zeiss MicroImaging GmbH, Jena, Germany) equipped with a multiline Ar-Laser (458/488/514nm), a DPSS-561 10 (561 nm), a Laser diode 405-30 CW (405 nm), and an HeNe laser (633 nm). Fluorescence images were taken by a Zeiss plan-Apochromat ×63 NA/1.4 oil-immersion. Intensity was measured as described in our previous work^58^ using Fiji 2.0 v1.51k and Igor Pro 6.36. Briefly regions of interest were determined using a variance filter (kernel 8 pixels), followed by a binary threshold and the particles analyze routine from Fiji. Image processing was performed with Photoshop CS4 (Adobe, Mountain View, CA).

### Phototoxicity studies and cell irradiation

#### Treatment and irradiation

Cells were inoculated (20 × 10^3^) into 96-well plates and left to incubate in complete media containing 10% FBS for 24 h. Cells were then treated with media only, 4-OHT (15 μM, post-irradiation unless otherwise specified), HYP (2 μM, 4 h) and HYPERTAM (HYP and 4-OHT). The DMSO content was at all times kept ≤0.25%. Following cell incubation with HYP for 4 h, all treatment groups were washed twice. The cells were irradiated from the plate underside by means of a *Lumisource* lamp (PCI Biotech AS, Oslo, Norway) through a 530 nm cut-off longpass filter (Roscolab Ltd, London, UK), at an irradiance of 4 mW/cm^2^. In the combination treatment experiments, 4-OHT (15 μM) was added again to all appropriate cell groups after irradiation.

#### Cytotoxicity assessment

The redox ability of all cell groups and dark controls was assessed by the MTT assay 24 or 48 h post-irradiation. At these time points the MTT assay outcome was utilized to estimate cell viability. The assay was performed by replacing cell media with complete media containing 0.5 mg/mL MTT and incubating at 37 °C in a 5% CO_2_ humidified atmosphere for 3 h. MTT media were subsequently aspirated from all cells and the produced formazan crystals solubilized with 100 μL DMSO per well. The plates were shaken for 10 min at ~300 r.p.m. in a Heidolph Titramax 101 orbital shaker (Heidolph Instruments GmbH & Co.KG), and the endpoint absorbance measurements at 570 nm were performed in a BioTek PowerWave XS2 plate reader (BioTek Instruments, Inc.). Blank values measured in wells with DMSO and no cells were in all cases subtracted.

### LDH leakage assay

Cells seeded in 35 mm Petri dishes (5 × 10^5^) were left to incubate in complete media containing 10% FBS for 24 h. The cells were subsequently incubated with HYP (2 μM) for 4 h washed, placed in OPTIMEM (Life technologies, 2 mL/dish), irradiated, and then 15 μM 4-OHT was added post-irradiation to the combination treatment groups. At 6 h after treatment, cell media (supernatants) were removed from all cell groups and placed on ice. The media-control cells were further washed with PBS, trypsinized, and centrifuged. The resulting cell pellet was resuspended in 2 mL OPTIMEM containing 0.25% Triton X-100 and agitated for 30 s. The resulting suspension constituted the 100% death control. An assay reaction mixture (pH = 7.4) was prepared containing 100 mM Tris-HCl, 0.4 mg/mL NADH, and 20 mM sodium pyruvate. Seven hundred microliters of the mix were added to 700 μL of sample (all collected supernatants; media-control cell lysate which signified 100% death; OPTIMEM only control) in a 1 mL cuvette with 1 cm light path length. The decay absorbance kinetics of NADH oxidation to NAD^+^ through pyruvate conversion to L-lactic acid by LDH were in each case monitored at 340 nm (*ε*_NADH 340nm_ = 0.00622 L μmol^−1^ cm^−1^), using a Shimadzu UV-2550 UV-VIS spectrophotometer (Shimadzu Corp.).

### Clonogenic assays

Four thousand cells were inoculated per 35 mm Petri dish (three dishes per treatment group) and 24 h later were treated with the PDT and 4-OHT protocols as described above. 4-OHT was removed from all relevant treatment groups 24 h post-irradiation and fresh media were added to all treatment groups. Approximately 2 weeks later (or when the colonies were optimal), the cells were fixed with ethanol for 10 min, and stained with crystal violet (0.05%). The cells were washed and the stained colonies containing more than 50 cells were counted in each group.

### ER knockdown

10^5^ cells/well were seeded in 12-well dish for transfection the next day. Pools of the following Silencer Select siRNAs were used (Invitrogen) s303873, n303876, s4826, s4827; 20 pmole each, with RNAiMax transfection agent (invitrogen), following the manufaturer’s instructions.

#### Immunoblots

Cells (~10^6^) were harvested at 6 h following irradiation of groups treated with vehicle, 4-OHT (post-irradiation, 15 μM, 4 h), HYP-PDT (2 μM, 4 h), or 4-OHT (post-irradiation) + HYP-PDT. The lysates were run on NuPage Bis Tris (LC3B) or Novex Tris Gly (ESR) gels. The proteins were subsequently transferred from the gels onto PVDF (LC3B) or nitrocellulose (ESR) membranes, using a Trans-Blot® Turbo™ transfer system (Bio-Rad Laboratories Inc.) (LC3B) or 50 mM Tris, 380 mM Gly, 0.1% SDS, 20% EtOH (ESR). Antibodies used were LC3B 1:2000 (CST 2775S), ESR2 1:1000 (Invitrogen PA1-311), ESR1 1:1000 (MA3-310), γ-Tubulin 1:20,000 (Sigma T6557). Signals were detected using a ChemiDoc™ MP Imaging System and intensities were quantified using Image Lab software (Bio-Rad Laboratories Inc.).

### Lipid peroxidation assay

We performed lipid peroxidation investigations using the Click-iT® lipid peroxidation detection with linoleamide alkyne (LAA; Life technologies Inc.). In brief the cells were co-incubated with 50 μM LAA and 4-OHT (overnight the previous night, 15 μM), HYP (2 μM), or 4-OHT overnight + HYP for 4 h, irradiated, and left for 1 h. LAA oxidation led to alkyne modifications at the nucleophilic side chains of proteins. These proteins could then be labeled by Alexa Fluor 488 azide, employing copper-catalyzed click chemistry upon cell fixation (4% formaldehyde, 15 min) and permeabilization (Triton X-100 0.5%, 10 min). Consequently, the cells were studied by confocal fluorescence microscopy (vide supra).

### Metabolic studies

In order to study the changes in cell metabolism under the insult of 4-OHT (15 μM), HYP (2 μM), and combination (4-OHT + HYP) with and without irradiation, we employed the Seahorse XF^e^96 analyzer (Seahorse Bioscience, Copenhagen, Denmark). Cells treated with 4-OHT (overnight), HYP (3h), or combination, had their media changed to XF base medium minimal DMEM (Seahorse Bioscience) supplemented with 10 mM glucose, 2 mM l-glutamine, 2 mM sodium pyruvate and adjusted to pH 7.4. 4-OHT and HYP were maintained in the appropriate groups and the cells were incubated for an extra hour at 37 °C, 0% CO_2_ (total 4 h). The appropriate cell groups were then irradiated while an identical plate was used as dark control. The basal oxygen consumption and media acidification rates (OCR and ECAR respectively) of the cell groups were measured in four cycles. The four corner wells were left devoid of cells and were used as blank values. These were automatically subtracted by the Seahorse analyser software.

All values obtained were normalized per cell line according to the DNA content. After the Seahorse measurements 25 μL of Quant-iT™ PicoGreen ® was added to 6 mL of TE buffer (10 mM Tris-HCl, 1 mM EDTA, pH 7.5). Seahorse media were aspirated and replaced with 50 μL of picogreen-TE buffer. The cells were incubated for 30 min at 37 °C, 5% CO_2_ and the endpoint fluorescence measurements were read at a BioTek Synergy 2 microplate reader (*λ*_ex_ = 480 nm, *λ*_em_= 525 nm). Blank values were obtained from wells with no cells yet treated with picogreen-TE and were subtracted from all cell group values.

### Flow cytometry analyses for apoptosis

MCF7 and MDA-MB cells 231 were seeded in 60 mm Petri dishes (6 × 10^5^ per dish) and left overnight at 37 °C and 5% CO_2_ humidified atmosphere. The cells were subsequently incubated with media only or HYP (2 μM, 4h). The cells were then washed twice with PBS and then the HYP-PDT and HYPERTAM groups were irradiated with the Lumisource lamp as detailed earlier. 4-OHT was subsequently added to the 4-OHT and HYPERTAM groups (15 μM). After 3, 8, and 24 h the cells were then subjected to two different apoptotic flow cytometry analyses for (A) Annexin V and (B) the TUNEL assay (8 and 24 h).

A. For Annexin V staining the cells were at the three selected time points trypsinized and stained for 30 min with Annexin V-FITC (5 μL/mL) and LIVE/DEAD IR (1 μL/mL) as per the manufacturers’ instructions. The cells were next washed with and resuspended in PBS containing Ca^2+^ and Mg^2+^. The flow cytometric analyses were performed using a CytoFlex S (Beckman Coulter, Brea, CA, USA) on a total of 20,000 events for each sample. FITC fluorescence was excited at 488 nm and registered after a bandpass filter at 525/40 nm. LIVE/DEAD IR fluorescence was excited at 638 nm and emission was collected after a 780/60 nm bandpass filter.
B. For the TUNEL (terminal deoxynucleotidyl transferase biotin dUTP nick end labeling) assay, the cells were initially trypsinized at the selected time points, and fixed with ice cold (−20 °C) methanol for at least 1 h (100%, 1 mL). Subsequently the cells were incubated in a TdT reaction mixture (TdT enzyme 0.2 μL, TdT reaction buffer 5 μL, CoCl_2_ 3 μL, biotin-16-dUTP 0.5 μL, and DTT 0.5 μL of 10 mM aqueous stock, H_2_O 40.8 μL—30 min at 37 °C), centrifuged (500 × *g*, 5 min), supernatant removed and pellets resuspended and incubated in 4% dry milk suspension containing streptavidin-AlexaFluor 488 (1:50 dilution, 0 °C, 30 min). cells were again centrifuged and resuspended in PBS containing Hoechst 33258 (1.5 μg/mL) for 30 min, RT. The flow cytometric analyses were performed using a LSRII (BD Biosciences, Franklin Lakes, NJ, USA) until at least 10,000 events were reached for each sample. AlexaFluor 488 fluorescence was excited at 488 nm and registered after a longpass filter with a cut-on at 505 nm and a bandpass filter at 530/30 nm. Hoechst 33258 was excited at 355 nm and the emission was collected after a 450/50 nm bandpass filter.

All flow cytometry data were analyzed using the FlowJo v.7.6.1 software (Treestar Inc., Ashland, OR, USA).

### Animal studies

For the HYPERTAM in vivo pilot study, inhouse bred, female NOD-scid Il2rγ^null^ (NSG) immunocompromised mice were employed. The mice were inoculated subcutaneously (i.p.) with 7.5 × 10^5^ cells of either MCF7 or MDA-MB-231, suspended in equal volumes of PBS and Matrigel, when they reached 6 weeks of age. After approximately 2 weeks for MDA-MB-231 and 3 weeks for MCF7, the tumor volume reached ~100 mm^3^, at which time the experiments could commence. The tumor volumes (mm^3^) were calculated with the help of the following formula: ${\mathrm {tumour}}\,{\mathrm {volume}} = \frac{{({\mathrm {longer}}\,{\mathrm {dimension}}) \times ({\mathrm {smaller}\,{\mathrm {dimension}}})^2}}{2}$ following caliper measurements twice weekly and reported as percentage change after treatment. When volume exceeded 1000 mm^3^, or mice showed clinical signs of cachexia and/or weight loss more than 20%, they were sacrificed by cervical dislocation under general anesthesia. Animals were allowed to eat and drink water ad libidum, while for the estrogen-dependent MCF7 tumors, the water was supplemented with estradiol (E2) at 6.25 μg/mL.

For the animal studies six groups of mice were used: control mice (CTRL) that were not administered any drugs or subjected to irradiation, HYP dark (HD) mice which were administered a full dose of HYP but were not exposed to light, TAM only (TAM) which were administered a full tamoxifen dose, HYP and TAM dark (HTD) which were administered full HYP and TAM doses but not light, HYP-PDT group (HL) which were administered PDT and then exposed to light 2 h later, and finally HYP-PDT + TAM which were administered HYP, exposed to light 2 h later, and then administered TAM. Each mouse group contained at least five mice while the groups of particular interest (HL and HTL) contained either six or seven mice.

From preliminary toxicology and PDT studies (data not shown), the optimal treatment regime was determined as follows: 12.5 mg/kg HYP was administered i.p., 2 h prior to irradiation (40 J/cm^2^) with the PDT lamp. Following PDT, the HTD, TAM and HTL groups were also dispensed one i.p. bolus of 200 mg/kg TAM. Both HYP and TAM formulations were achieved by initial dissolution in cremophore (15%) and subsequent dispersion in water (85%). The animals were anesthetized during the PDT process using gas anesthesia (sevoflurane), while all other skin areas but the tumor with a 1–2 mm peripheral margin were appropriately shielded from light. All animal experiments were executed in compliance with institutional, national, and European guidelines and regulations and after approval from the institutional review board of Radium Hospital (application number: 7489).

### Reporting Summary

Further information on research design is available in the Nature Research Reporting Summary linked to this article.

## Supplementary information

Supplementary information

Reporting summary

## Data Availability

The HPYERTAM efficacy and animal study survival data generated and analyzed during this study are described in the following data record: 10.6084/m9.figshare.7863128. As outlined in the data record, raw data are available from the authors on request.

## Supplementary information

**Supplementary Information** accompanies the paper on the *npj Breast Cancer* website (10.1038/s41523-019-0108-8).
