# Supplementary material for: Simultaneous defeat of MCF7 and MDA-MB-231 resistances by a hypericin PDT–tamoxifen hybrid therapy
Source: NPJ Breast Cancer. 2019 Apr 10;5:13. doi: 10.1038/s41523-019-0108-8 (PMC6458138; doi:10.1038/s41523-019-0108-8)
Supplement: Supplementary file 1 — Supplementary information [file 41523_2019_108_MOESM1_ESM.pdf]

SUPPLEMENTARY INFORMATION

Simultaneous defeat of MCF7 and MDA-MB-231 resistances by a hypericin PDT–tamoxifen hybrid therapy

Theodossis A. Theodossiou[1]*, Muhammad Ali[2], Mantas Grigalavicius[1], Beata Grallert[1], Pierre Dillard[3], Kay Oliver Schink[4], Cathrine E. Olsen[1], Sébastien Wälchli[3], Else Marit Inderberg[3], Andreas Kubin[5], Qian Peng[6] and Kristian Berg[1].

.

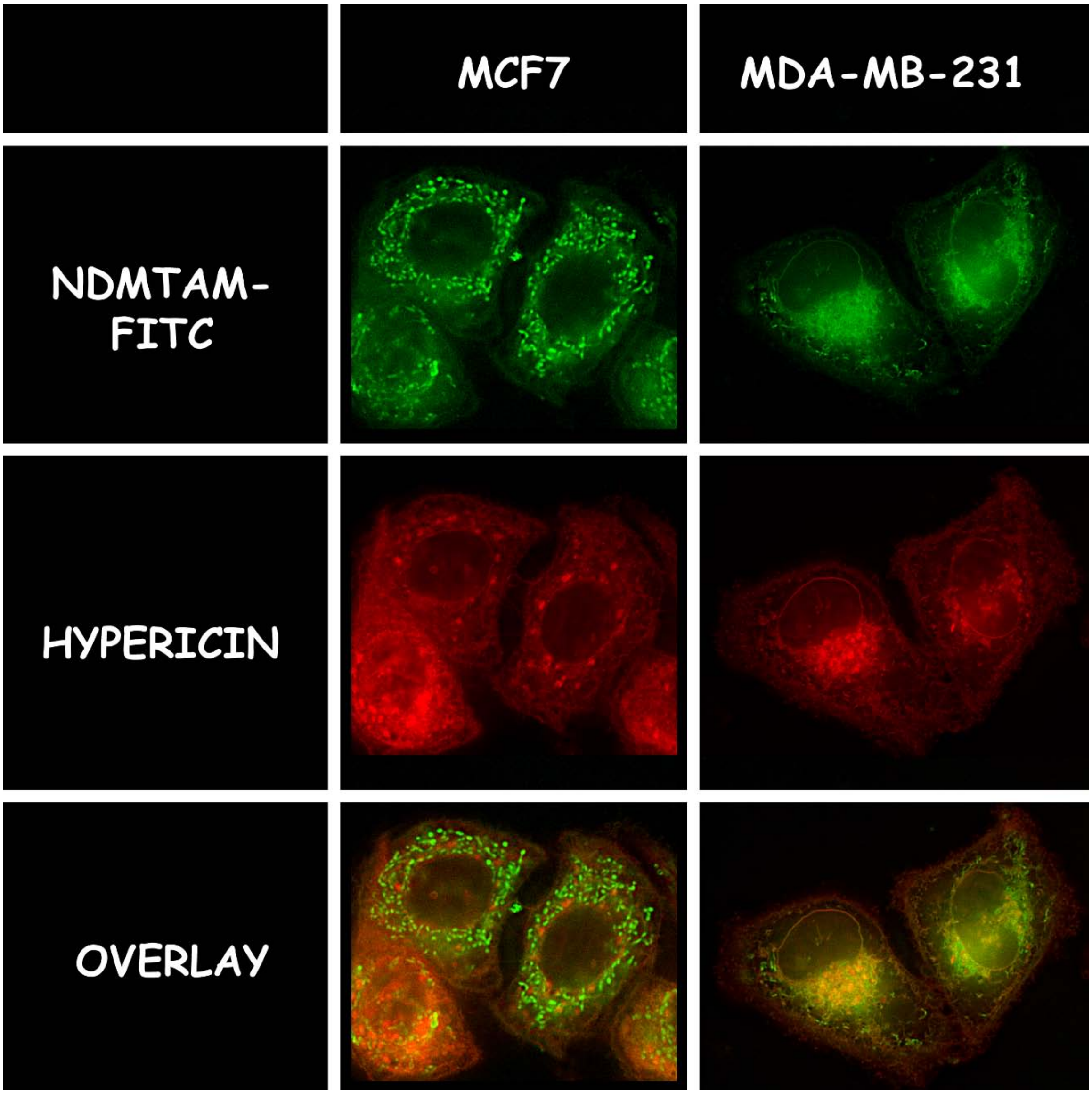

Supplementary Figure 1. Intracellular co-localization studies of HYP and FITC-conjugated *N*-desmethyltamoxifen (NDMTAM). HYP (2 μM) and NDMTAM (10 μM) were administered to MCF7 and MDA-MB-231 cells for 4h and then their intracellular patterns were studied by deconvolution microscopy. NDMTAM fluorescence is shown in green and HYP fluorescence in red, while co-localisation is shown in orange-yellow.

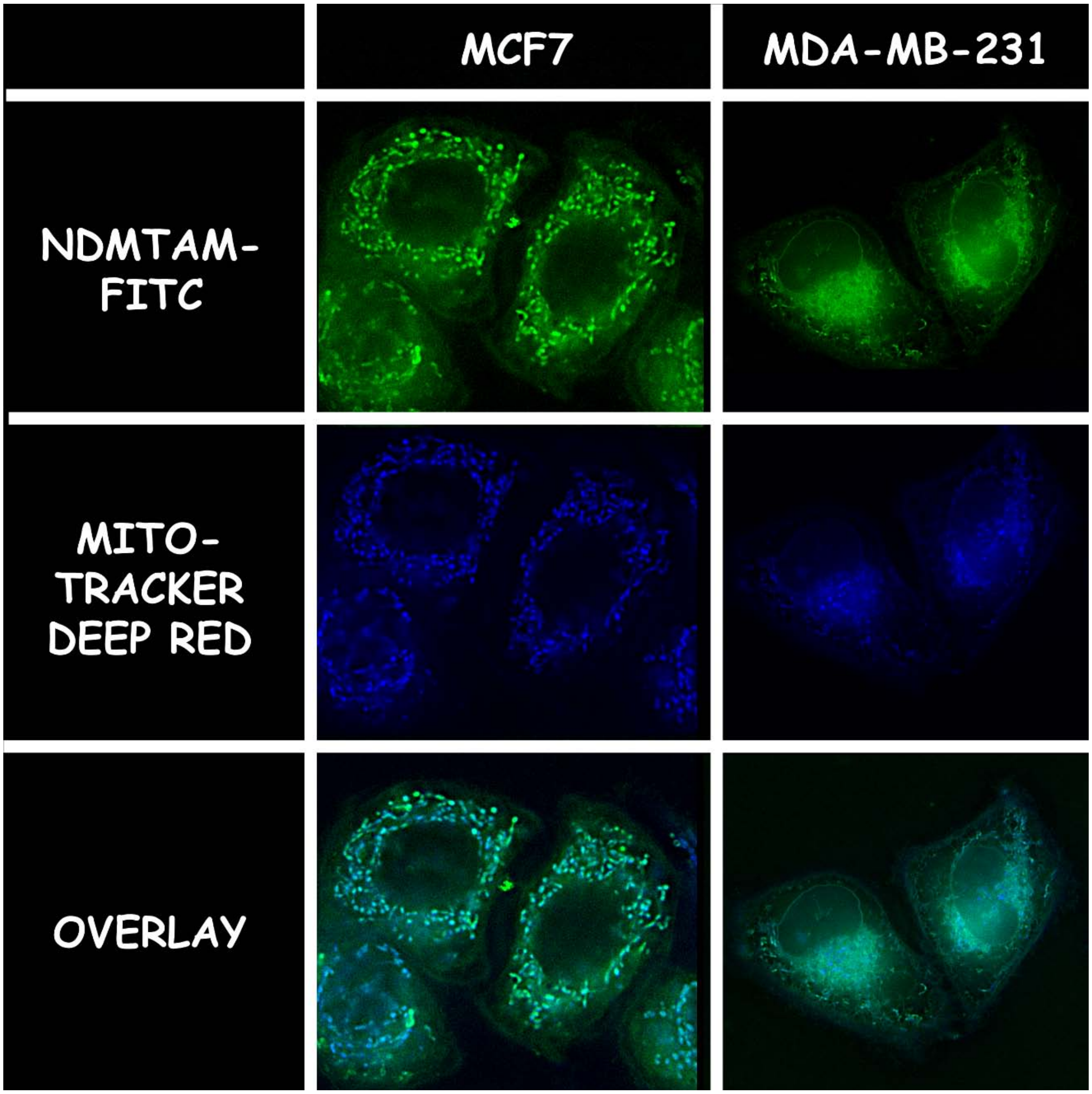

Supplementary Figure 2. Intracellular co-localisation studies of NDMTAM (10 µM, 4h) and Mitotracker Deep Red FM® (150 nm, 5 min), by deconvolution microscopy. NDMTAM fluorescence is shown in green and Mitotracker Deep Red FM® fluorescence in blue, while co-localisation is shown in cyan-white.

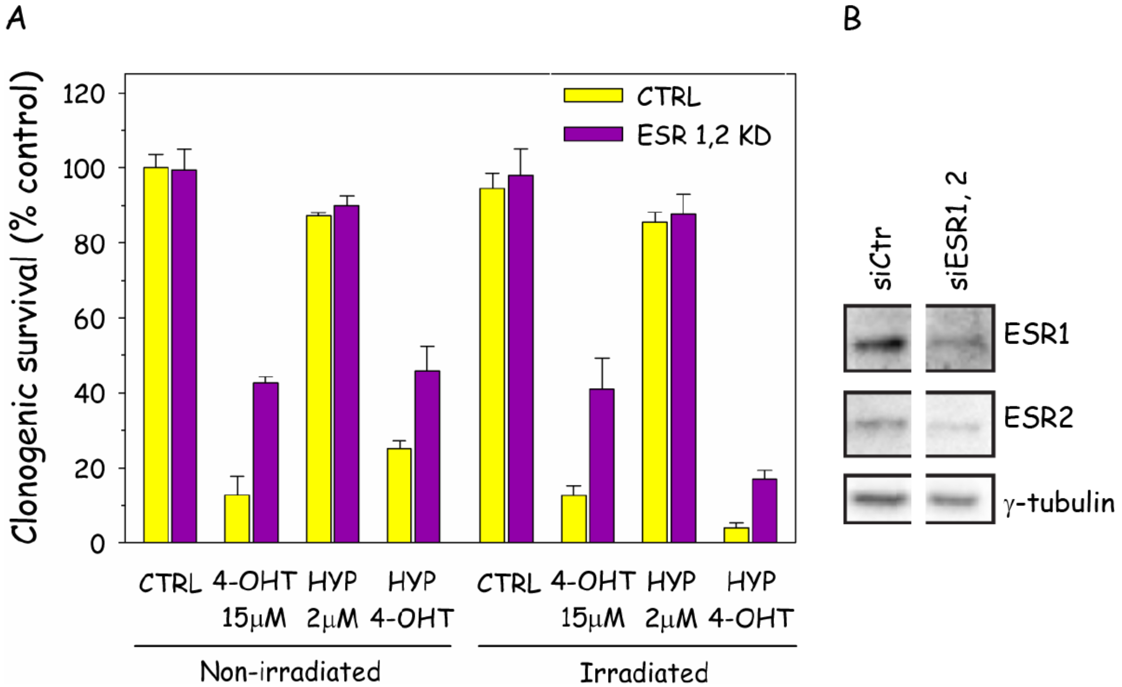

Supplementary Figure 3. Clonogenic survival after HYPERTAM upon knockdown of estrogen receptors (ESR) in MCF7 cells. A. Clonogenicity on MCF7cells, either treated with vehicle siRNA (CTRL, yellow bars) or SiESR1,2 (ESR1,2 KD, purple bars). B. representative blots of cells treated with either SiCtrl or SiESR1,2 on the 4th day following transfection when the HYP-PDT/HYPERTAM treatments were administered. The ESR1 depletion on that day, was found to be ~60%, while the ESR2 depletions was ~40%. Normalization was achieved with respect to the housekeeping gene γ-tubulin. The error bars represent STD from the mean of 3 parallels. All blots shown were derived from the same experiment.

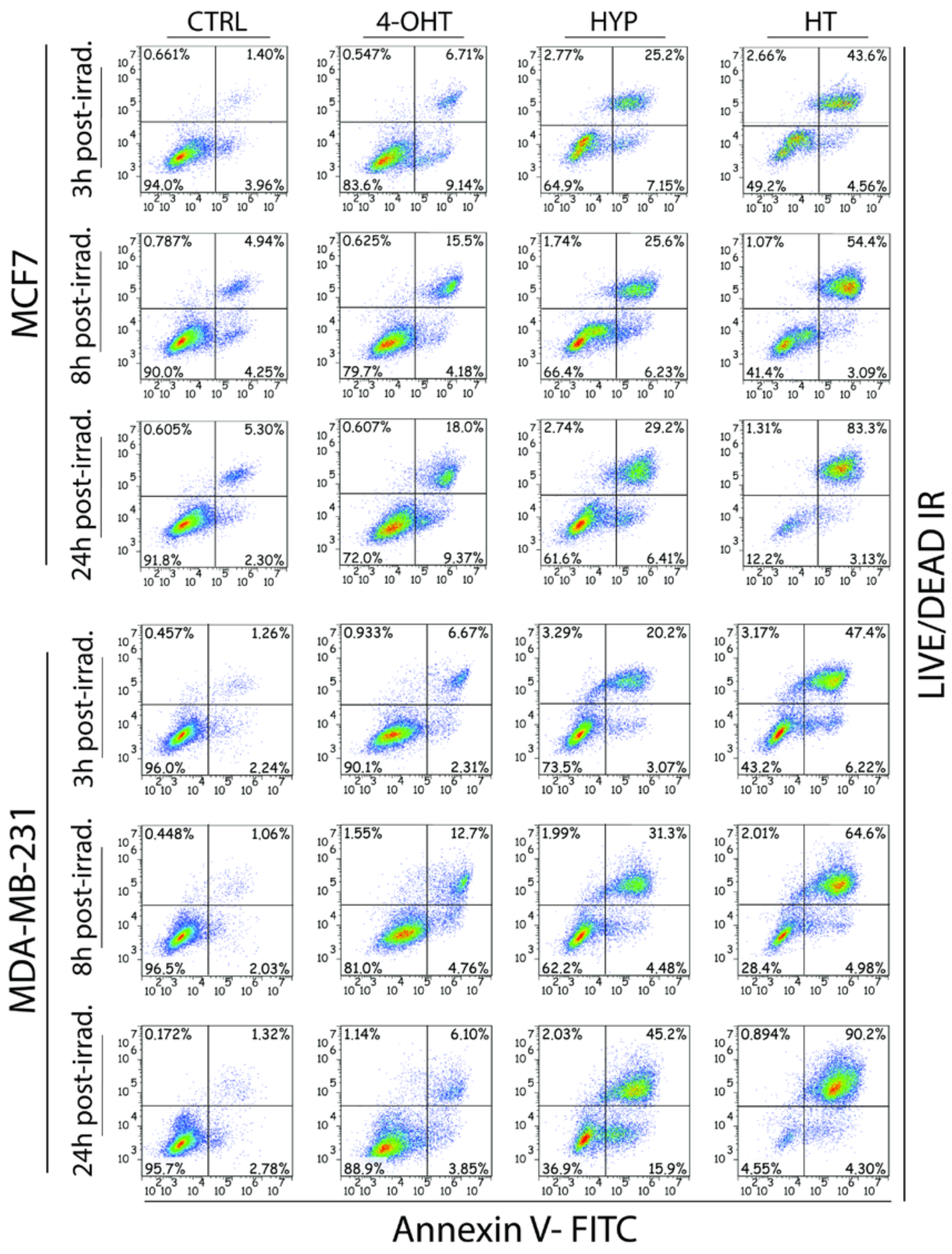

Supplementary Figure 4. Flow cytometry plots of MCF7 and MDA-MB-231 cells treated with media only (CTRL), 4-OHT, HYP-PDT (HYP) and HYPERTAM (HT). Flow cytometric analysis was carried out on live cells at 3, 8 and 24h post irradiation. The cells were stained with Annexin V-FITC and LIVE/DEAD IR.

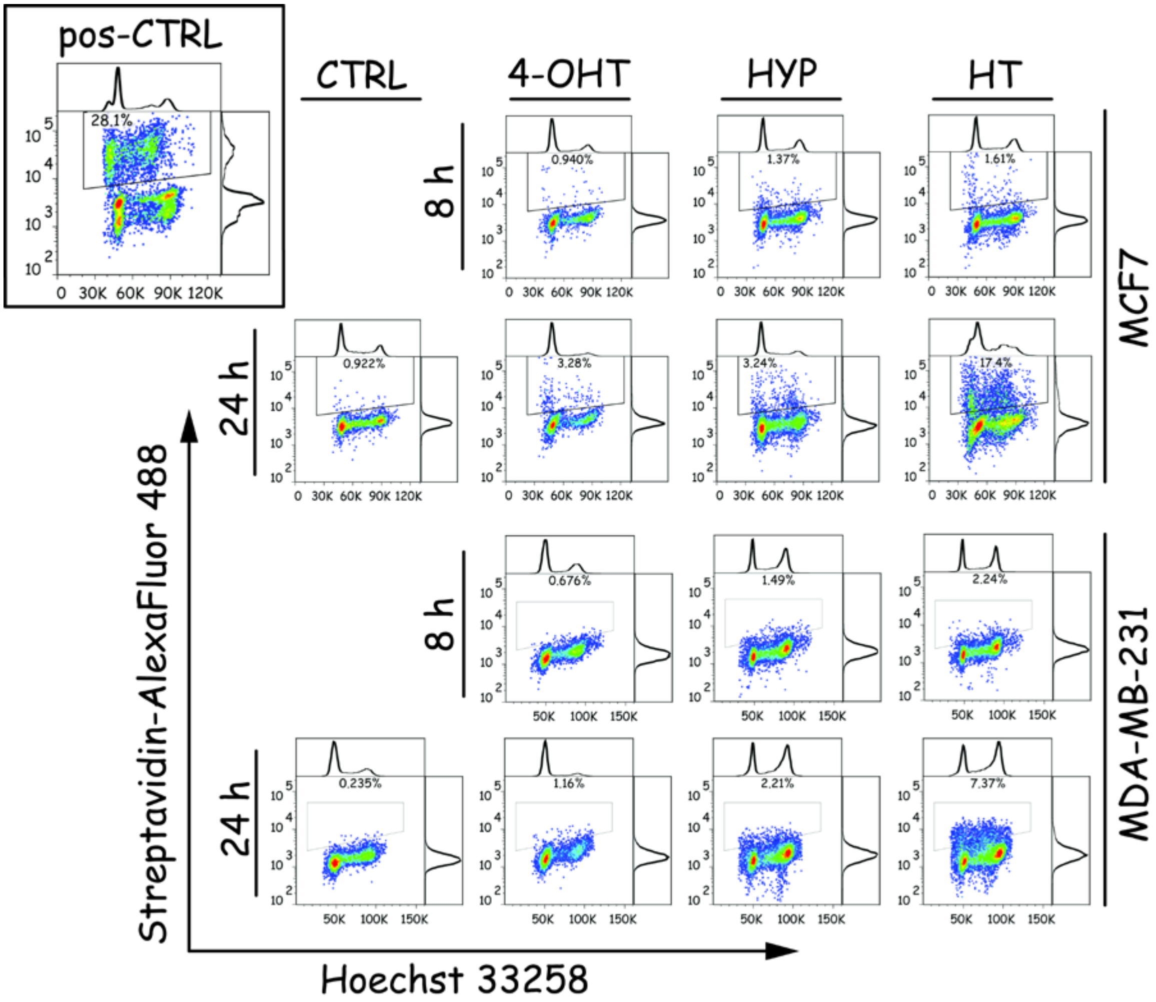

Supplementary Figure 5. Flow cytometry plots of MCF7 and MDA-MB-231 cells treated with media only (CTRL), 4-OHT, HYP-PDT (HYP) and HYPERTAM (HT). The fraction of cells undergoing apoptotic nuclei DNA fragmentation was investigated employing the TUNEL (Terminal deoxynucleotidyl Transferase Biotin dUTP Nick End Labeling). Cells were methanol fixed and permeabilized, at the designated timepoints, and the DNA fragmentation “nick ends” were labeled with AlexaFluor 488. Apoptotic gating in all plots is identical and adjusted to the CTRL. The positive control (top left corner) consists of B-lymphocyte REH cells exposed to 4 Gy, and harvested after 24h.

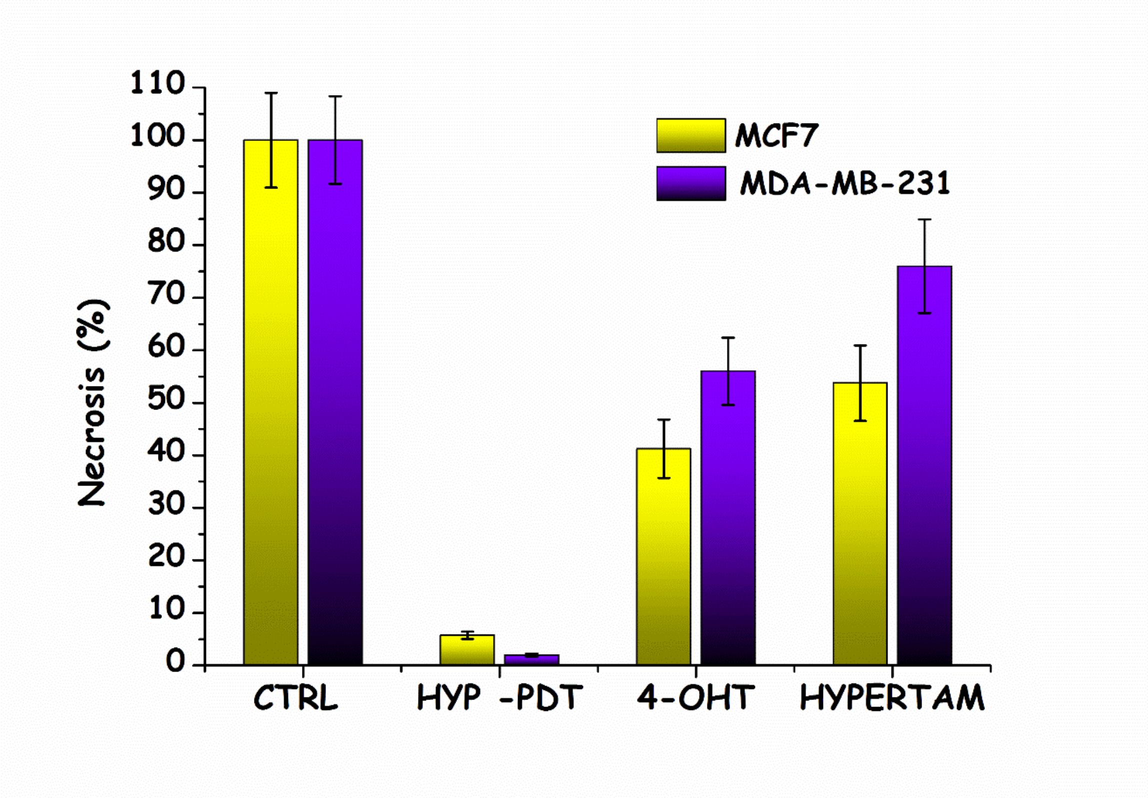

Supplementary Figure 6. Effects of HYPERTAM on cell necrosis. LDH leakage assays 24h post-treatment in MCF7 and MDA-MB-231 cells. The assay is based on the release of LDH from necrotic cells which can be measured by the decay of NADH absorbance at 340 nm with time, representing LDH-mediated oxidation of NADH to NAD^+, in the presence of pyruvate. The100% necrosis controls are obtained by LDH measurements in the supernatants of untreated (control) cell lysates. The error bars represent STD from the mean between 3 parallels.

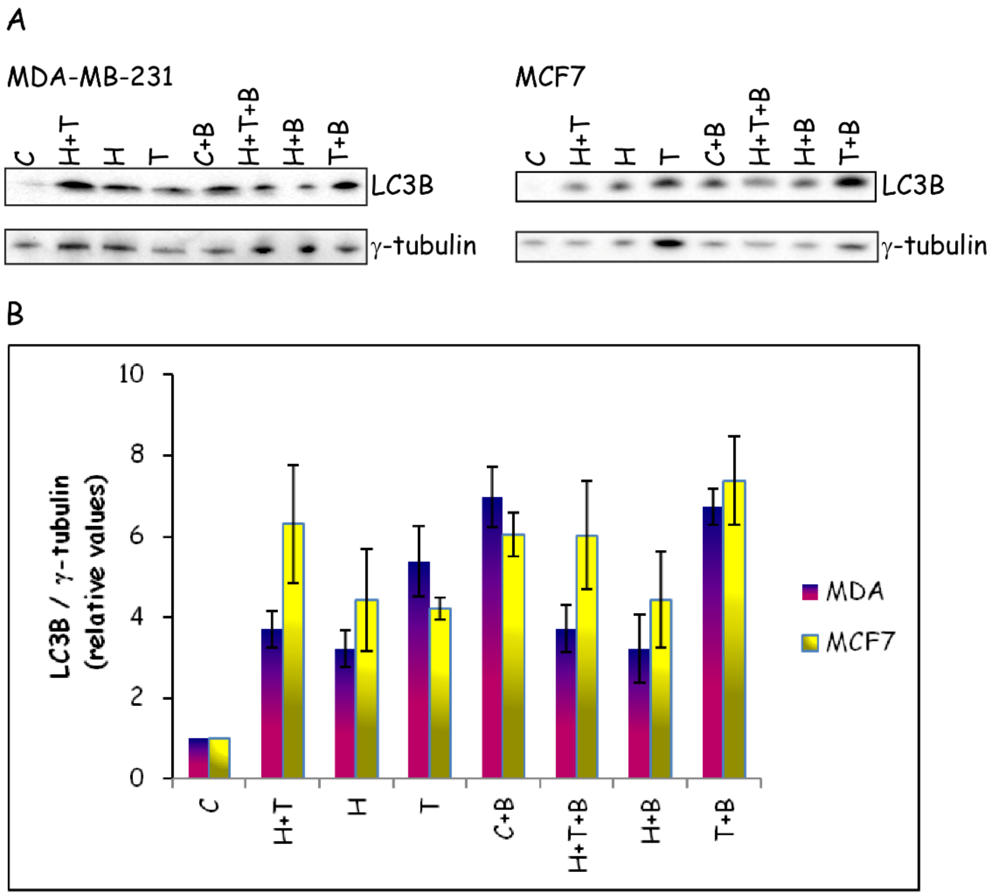

Supplementary Figure 7. Effects of HYPERTAM on cell autophagy A) Western blots for LC3B in MCF7 (right) and MDA-MB-231 (left) cells, as a marker of autophagy, 6h post irradiation. The blots were performed in media only control cells (C), 4-OHT treated cells (15 μM, 24h), HYP-PDT treated cells (H) and HYP-PDT + 4-OHT treated cells (HT). In addition. 50nM bafilomycin (B) was added after washing the cells, just before irradiation, with all combination as a positive control for autophagy. The house-keeping gene of choice was γ-tubulin. B) The results of 4 independent WB experiments for MCF7 and 5 WB experiments for MDA-MB-231, were quantified and the mean values ± SE are presented for all groups. All blots shown were derived from the same experiment.

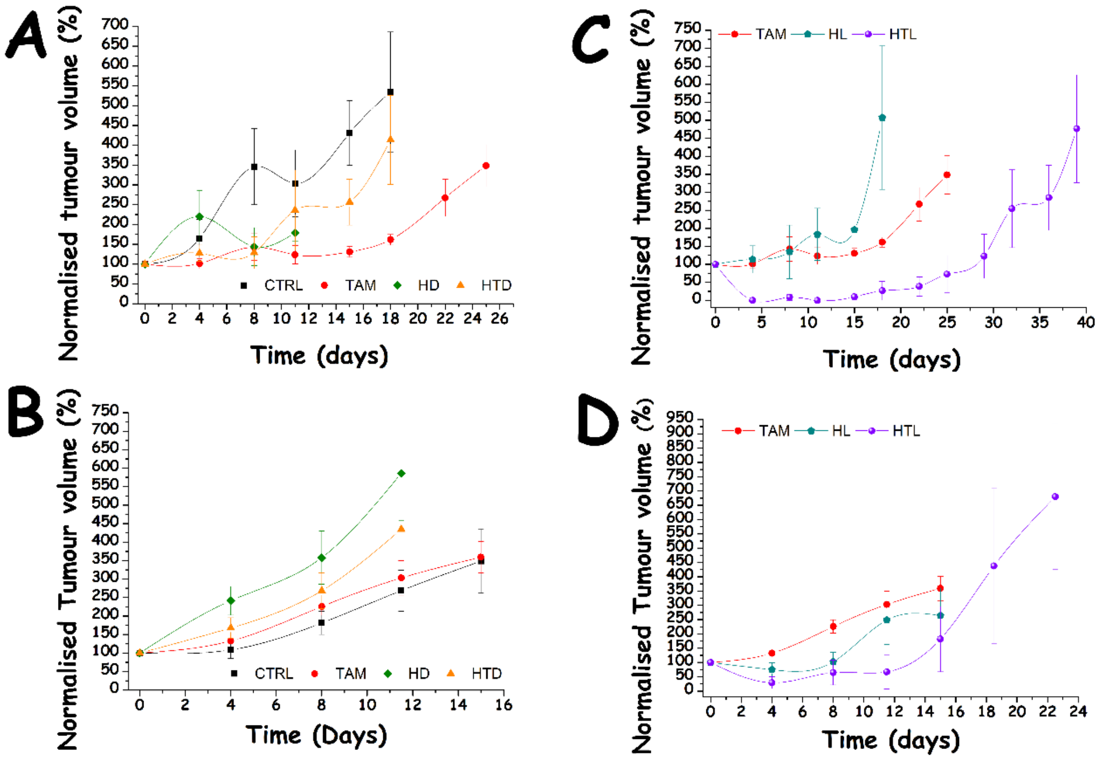

Supplementary Figure 8. Tumor growth for the different treatment groups of the MCF7 (A, C) and MDA-MB-231 B, D) tumor models. CTRL (untreated), HD (HYP dark), TAM (tamoxifen only), HTD (HYP+TAM dark), HL (HYP PDT), HTL (HYPERTAM). In these graphs the post-treatment tumor volumes are normalized to the pre-treatment values which were defaulted to 100%. The values shown are the mean values for each group until the first death occurrence. The errors bars in all cases represent the SEM values for n≥5.

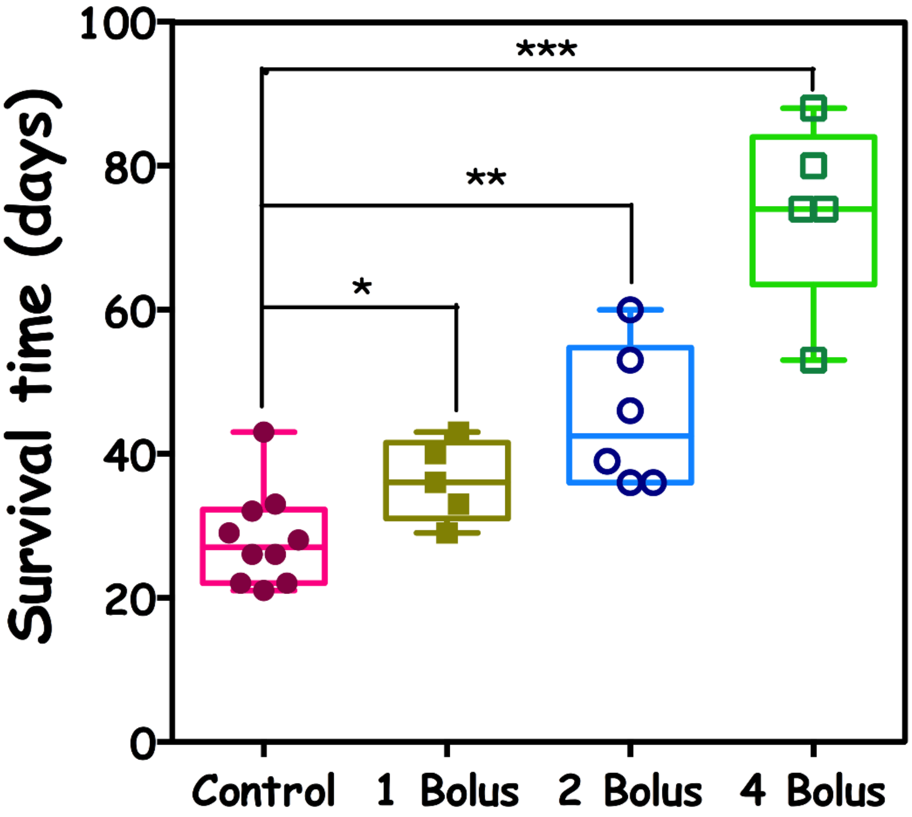

Supplementary Figure 9. Mice survival vs. number of tamoxifen boluses. NSG mice survival shown as data points around the median for different treatment groups of the MCF7 tumor models. CTRL (untreated, purple solid circles), 1 bolus of TAM (dark yellow squares), 2 boluses of TAM (one bolus per week, open blue circles) and 4 boluses of TAM (one bolus per week, green open squares). The TAM boluses (2 and 4) were identical to the ones used for the HYPERTAM treatment (Fig. 5 and S8). The data presented here were pooled from two separate experiments. The HYPERTAM experiment (Fig. 5A) contained a control and a 1 bolus groups, whereas the second, supplementary, experiment contained a control, a 2 boluses and a 4 boluses groups. The statistical significance was calculated using a one tailed student t-test; ns $P \geq 0.05$, * $P<0.05$ and ** $P<0.01$, *** $P<0.001$. The whiskers signify min and max values.

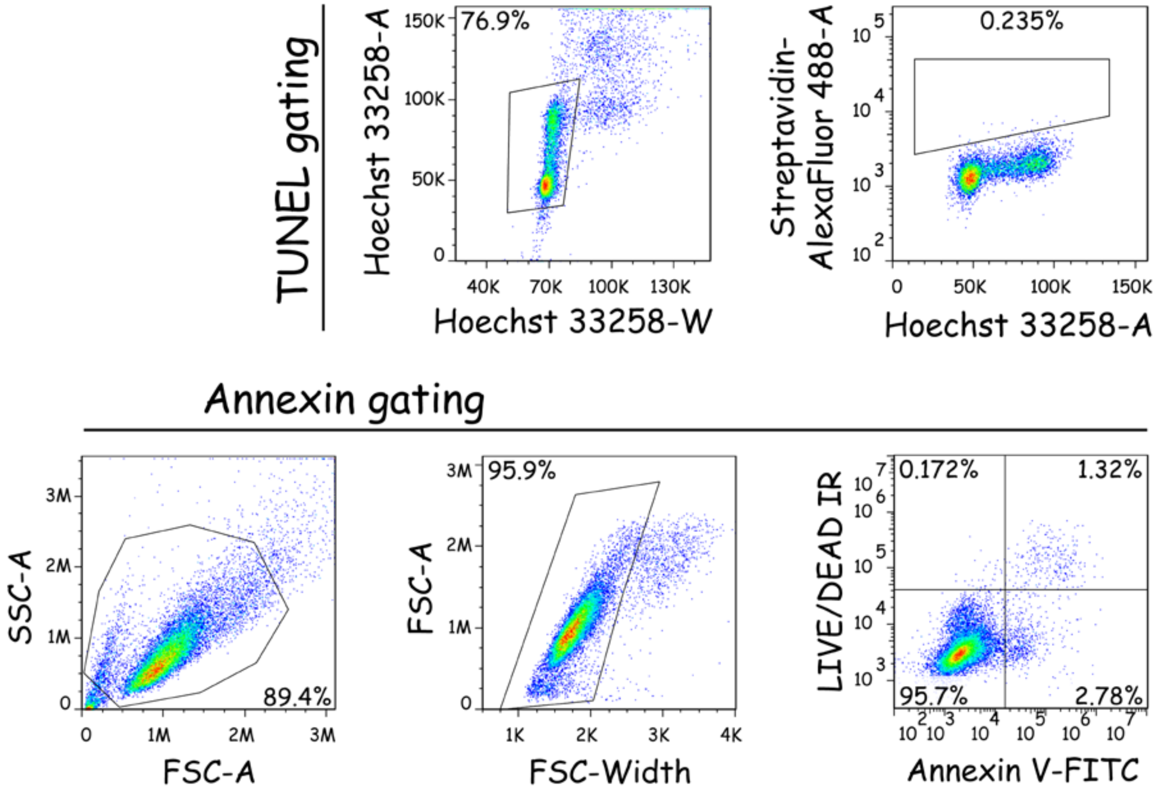

Supplementary Figure 10. Gating strategy used in the analysis of the flow cytometry data presented in Fig. S4 (Annexin V vs LIVE-DEAD) and Fig. S5 (TUNEL assay).

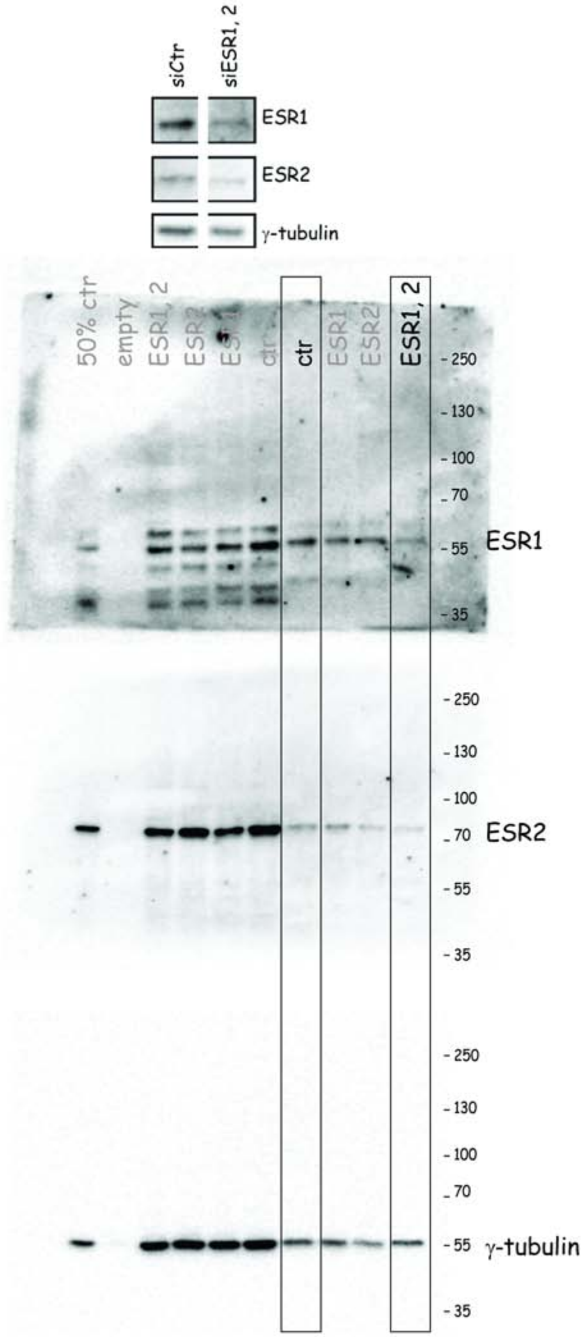

Supplementary figure 11. The upper panels show the cropped immunoblots as they appear in Fig S3B. The lower panels show the non-cropped blots with size markers. The rectangles highlight the bands shown in Fig S3B.

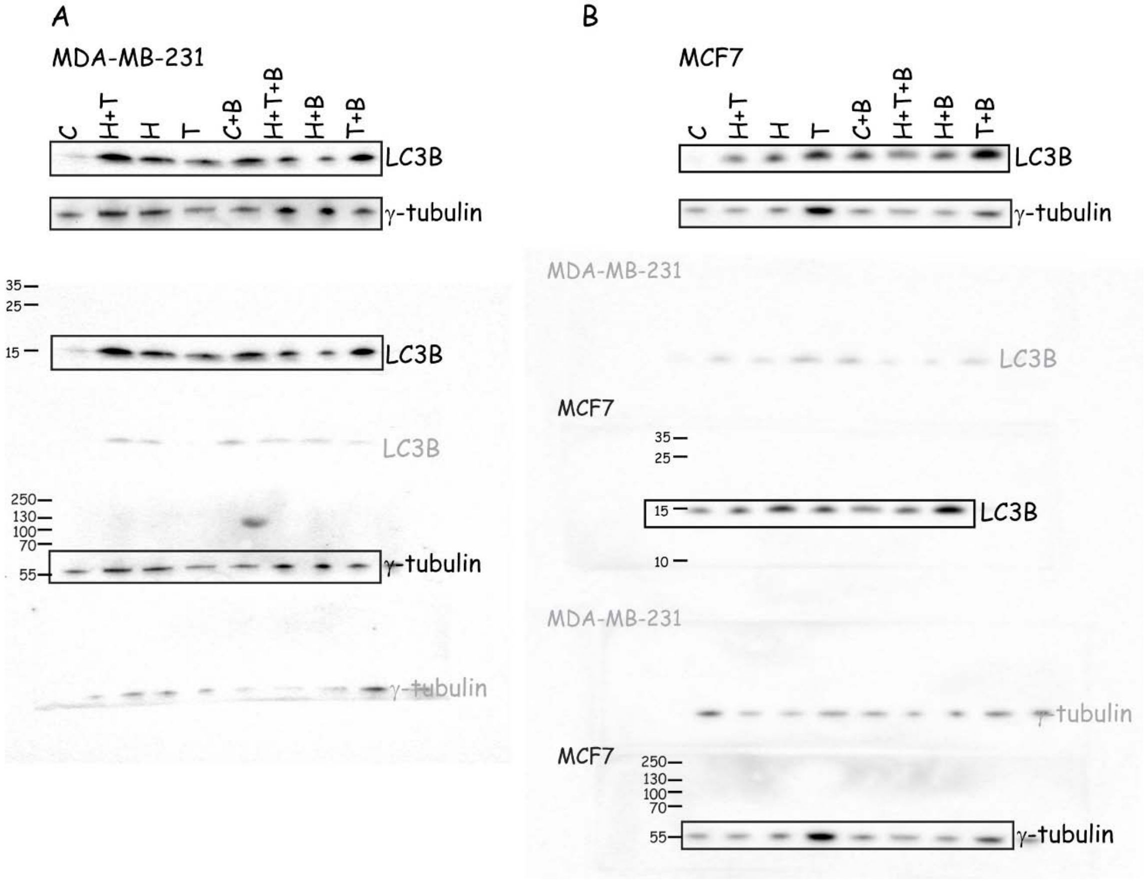

Supplementary figure 12. The upper panels show the cropped immunoblots as they appear in Fig S7A. The lower panels show the non-cropped blots with size markers. The samples from MCF7 (A) and MDA-MB-231 (B) cells were run on two separate gels. The blots were cut at 35 kDa. The upper halves were probed for γ-tubulin, the lower halves for LC3B. Therefore the non-cropped images show four strips, two and two from the same gel. The rectangles highlight the bands shown in fig S7A.

Supplementary Table 1 Median mice survivals for the various treatment groups. CTRL (untreated), HD (HYP dark), TAM (tamoxifen only), HTD (HYP+TAM dark), HL (HYP PDT), HTL (HYPERTAM). Significant differences between the two cell lines are highlighted red.

	**Median survival (days)**	
**Treatment groups**	**MDA-MB-231**	**MCF7**
CTRL	28	26
TAM	22	36
HD	23	21
HTD	23	35
HL	36	28.5
HTL	37	53
